# Simple Physical Model for the Estimation of Irreversible Dissociation Rates for Bimolecular Complexes

**DOI:** 10.1021/acs.jpca.3c01890

**Published:** 2023-07-07

**Authors:** Lauri Franzon

**Affiliations:** Department of Chemistry, University of Helsinki, A.I. Virtasen aukio 1, P.O. Box 55, 00014 Helsinki, Finland

## Abstract

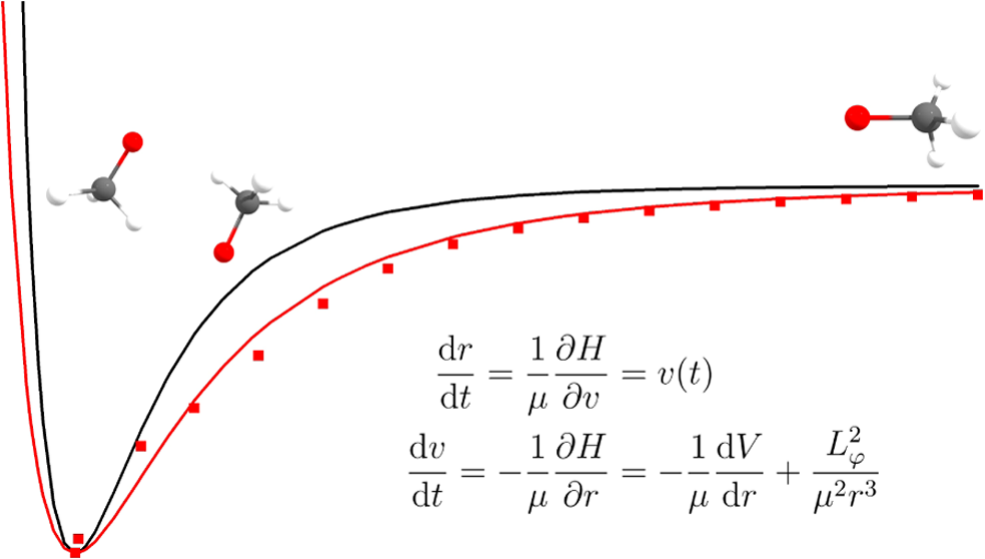

In this article, we propose a simple method of estimating dissociation rates of bimolecular van der Waals complexes (“wells”), rooted in rigid body dynamics, requiring as input parameters only the bimolecular binding energy, together with the intermolecular equilibrium distance and moments of inertia of the complex. The classical equations of motion are solved for the intermolecular and rotational degrees of freedom in a coordinate system considering only the relative motion of the two molecules, thus bypassing the question of whether the energy of the complex is statistically distributed. Well-escaping trajectories are modeled from these equations, and the escape rate as a function of relative velocity and angular momentum is fitted to an empirical function, which is then integrated over a probability distribution of said quantities. By necessity, this approach makes crude assumptions on the shape of the potential well and neglects the impact of energy quantization, and, more crucially, the coupling between the degrees of freedom included in the equations of motion with those that are not. We quantify the error caused by the first assumption by comparing our model potential with a quantum chemical potential energy surface (PES) and show that while the model does make several compromises and may not be accurate for all classes of bimolecular complexes, it is able to produce physically consistent dissociation rate coefficients within typical atmospheric chemistry confidence intervals for triplet state alkoxyl radical complexes, for which the detailed balance approach has been shown to fail.

## Background and Introduction

Short-lived intermediate compounds are a subject of interest in fundamental reaction chemistry, particularly when determining branching ratios for reactions in kinetically controlled conditions. This work is concerned with a particular type of short-lived intermediate that is quite important, for example, in gas-phase atmospheric chemistry: a weakly binding bimolecular van der Waals complex with so-called roaming reactions^1^ fast enough to compete with irreversible dissociation. If the product molecules (A and B in Scheme 1) are sufficiently unstable, these intermediates are exclusively formed in dissociations of a precursor compound, not from bimolecular association.
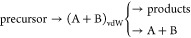
1

Typically, the rate coefficient of the dissociation pathway (A + B)_vdW_ → A + B *k*_d_ is determined using the detailed balance method, which assumes that the rate coefficients of association and dissociation correspond to those determinable in conditions close to thermal equilibrium. This is a convenient model to use as simple kinetic models of bimolecular association are more abundant than models of dissociation. When combined with high-level quantum chemical calculations of the dissociation energy and variational transition state theory calculations for *k*_a_, this approach typically leads to excellent agreement with experimental measurements of *k*_d_ for strongly and moderately bound systems.^2,3^ In the canonical formulation of detailed balance, the rate of dissociation is determined by the equation
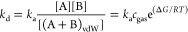
2where *c*_gas_ is the ideal gas concentration expressed in units consistent with the association rate coefficient *k*_a_, *R* is the ideal gas constant, *T* is the temperature, and Δ*G* ≡ *G*_(A+B)_ – *G*_A_ – *G*_B_ is the (molar) Gibbs free energy of dimerization. The original discussion on the merits of detailed balance was largely based on experimental observations of the kinetics of dissociation and association of diatomic molecules,^4^ and on the assumption that the rate of intramolecular relaxation of normal modes will always be significantly faster than chemical reactions depending on excitation of a single mode. Later, Smith et al.^5^ provided a derivation showing that the detailed balance assumption holds for bimolecular reactions in which the association goes through a van der Waals complex. This is somewhat closer to our interests, but the argumentation relies on the reversibility of the A + B ↔ (A + B)_vdW_ step, assuming a stable background concentration of A and B, which is not the case if one or both molecules are unstable. Finally, Miller and Klippenstein^6^ convincingly argued for the detailed balance using a master equation formalism, showing that the detailed balance assumption should always apply if the eigenvalues of association and dissociation are separable from the continuum of internal energy relaxation eigenvalues. It does not require that the majority of dissociation occurs after the thermal relaxation.

In Source (6) this discussion was framed entirely around a one-dimensional energy-resolved master equation approach, without going further into the physical origin of fast or slow energy relaxation. This is strongly related to the couplings between the vibrational modes in a molecule.^7^ A bimolecular complex has six intermolecular modes with generally low frequencies, and the vibrational energy flow through these has long been hypothesized to be slower than through intramolecular modes. Thus, the intermolecular energy relaxation might just be slow enough to break the applicability of detailed balance. A partially related subject is the matter of non-ergodicity, which occurs when the dissociation outspeeds the intermolecular energy relaxation. In this case, kinetic methodologies derived using statistical ensembles (the Eyring equation, RRKM, etc.) are all inaccurate, at least if the complex as a whole is treated as a system in equilibrium.^8^ It has long been well known that dissociation reactions with weakly bound complexes have this property, complexes with noble gas bonds being a particularly strong example.^9^ Marcus himself made a distinction between weakly and strongly bound complexes when discussing the applicability of the RRKM model for dissociation reactions.^10^ We are not aware of any systematic study of intermolecular relaxation rates of bimolecular complexes, but there exist numerous experimental studies of photochemical reactions of bimolecular complexes^11,12^ and *S*_*N*_2 reactions with bimolecular complexes as intermediates^13,14^ with nonstatistical product distributions, indicating that there are limiting conditions for when these complexes can be considered ergodic. Simple molecule + atom -complexes, on the other hand, show experimental results more in line with the ergodicity assumption.^15^ So, in short, the detailed balance assumption might be valid for irreversible dissociation in some cases, and it might be invalid in others. We do not know enough about their dynamics to accurately judge where the line goes.

There is a second practical problem for determining *k*_d_ for bimolecular complexes using detailed balance: Δ*G* is notoriously difficult to determine accurately for these systems.^16^ The binding energy, and thus the low-temperature limit of Δ*G*, can be determined computationally with reasonable accuracy,^17^ but the entropy contribution causes problems, especially due to the contribution of the six intermolecular vibrational modes, whose computational frequencies depend strongly on the method, and which are badly characterized by the harmonic oscillator model used as a first-order approximation in most quantum chemistry programs.^16^ Benchmark studies of room-temperature thermochemical properties of strongly binding (acid–base, ion–dipole) complexes^18,19^ and even H-bonded complexes^20^ have shown some success with advanced quantum chemical optimization while still using the rigid rotor harmonic oscillator approximation (RRHO) with frequency scaling factors and quasi-harmonic hindered rotor corrections for internal rotations,^21^ but likely this is more difficult for weakly bound complexes with shallow potential wells. Similar benchmark studies for acid cluster complexes^22,23^ with anharmonicities accounted for using a perturbative approach^24,25^ have resulted in modest improvements to the scaled harmonic frequencies. Going beyond perturbed RRHO, accurate modeling of the dimerization equilibrium constant for the water dimer^26^ from room temperature almost up to boiling point has been calculated by explicitly treating the system as a bimolecular complex,^27^ thereby including the coupling between rotational modes of the complex and the molecules in the Hamiltonian, as well as including the nonrigidity of molecules using an adiabatic model, and counting the rovibrational states below the dissociation energy using a Lanczos algorithm.^28^ While this is certainly a rigorous approach specifically tailored for modeling the intermolecular vibrational modes, it would most likely prove costly to implement this for larger bimolecular complexes. Finally, the existence of multiple local minima in the van der Waals well also contributes to the binding entropy of bimolecular complexes,^29^ which further complicates the accurate computational determination of Δ*G*.

We propose a slightly different method for determining irreversible dissociation rates for bimolecular complexes, rooted in rigid body classical mechanics, which sidesteps the issues related to calculating Δ*G* accurately. In terms of accurately modeling the relevant physics, “classical” is likely less of a problem than ’rigid,’ as dissociation of the complex requires excitation into the continuum of unbound quantum states, at which point the molecular motion should be reasonably well described by classical trajectories. Treating the molecules as rigid bodies compromises the accuracy a bit more, but the approach has three distinct advantages. First, the method only requires knowledge of the complex binding energy Δ*E* and some estimate of its dependence on intermolecular distance. We do not have to tackle the more difficult entropy of complex formation. Second, the approach circumvents the problem of ergodicity by using a simple mathematical trick often used for two-body systems. The kinetic energy of a two-particle system can be split into components of combined and relative motion
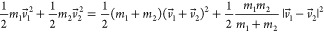
3

The dissociative motion is only dependent on the relative position and orientation of the two molecules, so we choose a Hamiltonian that only includes the relative position, momentum, and orientation of the two molecules. In this framework, the distribution of kinetic energy between the two molecules does not matter. This necessarily means that anharmonic coupling between intramolecular and intermolecular modes must be ignored, which is one of the largest compromises alluded to above. Finally, the third advantage is that rigid body classical mechanics allows us to perform large amounts of trajectory simulations with a very low computational cost. As the main question in atmospheric chemistry often is if a given reaction is competitive or not, a crude order-of-magnitude estimate is often enough.

### Atmospheric Background: Alkoxyl Radical Complexes

The main reason behind our choice of model is to model the dissociation of triplet state alkoxyl radical complexes, for which the detailed balance approach resulted in unphysically high dissociation rates,^30^ the fastest of which are higher than the intermolecular vibrational frequencies that the internal energy equilibration of the complex depends on. This is a paradox: calculating dissociation rates with a model assuming ergodicity results in a rate implying that ergodicity cannot physically apply. Whether this is due to limitations in the detailed balance approach, due to inaccuracies in Δ*E*, Δ*S*, or for some other reason has not been fully narrowed down.

Alkoxyl radical complexes form as unstable intermediate products of peroxyl radical recombination.^31^ The peroxyl radicals combine into a metastable tetroxide in the singlet state, which decomposes into a ground-state (triplet) molecular oxygen and a pair of alkoxyl radicals also coupled as a triplet, as shown in eq 4. This dissociation reaction is often endothermic,^32^ and the complex is thus ‘born cold.’ This means that nonthermal effects such as spin-flips^33^ and H-shift tunneling^30^ play a role in determining if the radicals react or dissociate. The complexity of the chemistry involved underlines the importance of determining physically accurate dissociation rates.
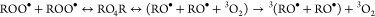
4

As this is the chemical problem that inspired this work, our test set exclusively consists of alkoxyl radical complexes, with computationally determined binding energies and geometries lifted from sources.^30,34−36^ The same approach may naturally be used for other systems, but we are currently not aware of other complexes for which (a) the irreversible dissociation rates are crucial enough to model with this detail, and (b) the detailed balance approach fails or proves cumbersome in this manner. Another possible atmospheric example of such a system is the dissociation of primary ozonides into a carbonyl and a Criegee intermediate, but in this case, the high excess energy of these complexes makes it unlikely that condition (a) is satisfied at typical atmospheric pressures.^37^ It should be noted that we are specifically using the density functional theory (DFT)-calculated binding energies from the above sources due to the apparent failure of CCSD(T)-F12 in producing chemically consistent binding energies for these compounds.^30^ Other than this, we will make no judgments on the feasibility of the binding energies, as the main focus of this work is on introducing our model.

## Methodology

### Equations of Motion

**Figure 1:**
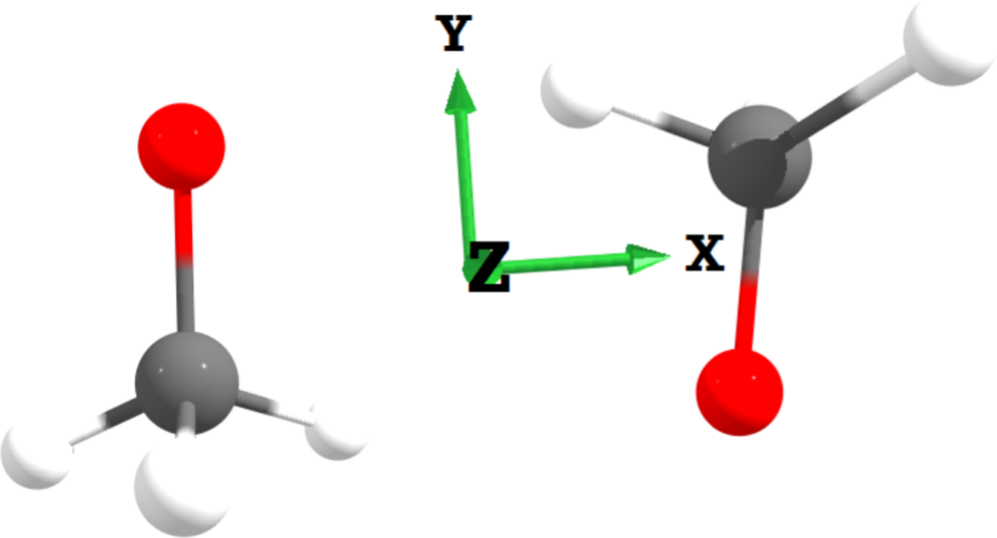
Three rotational axes of the (MetO)_2_ dimer. The axis labeled *X* is the principal axis, whereas *Y* and *Z* are the two rotational axes for which *I*_*i*_ ≈ μ*r*^2^. The ’torsional modes’ from the text are modes where only one molecule rotates around an axis. As seen in the Supporting Information, this approximation is reasonably accurate for the shown system.

Much like the most successful methods of determining the thermochemistry of van der Waals complexes,^27^ we will be focusing on modeling the six intermolecular vibrational modes and the rotational modes coupling to these explicitly, on the level of rigid body dynamics. First of all, we know these six modes are converted to free translational and rotational modes of the individual molecules in the dissociative limit *r⃗* → ∞, *r⃗* being the relative distance between the two centers of mass. As such, we will treat them as three translational modes and three torsional modes, both bounded by an intermolecular potential energy , where  is the relative orientation of the molecules. The coordinate system considered is one is one aligned with the three rotational axes (Figure 1) of the complex. As mentioned in the introduction, we are also neglecting the quantization of energy as we are only interested in unbounded states. As such, the classical Hamiltonian is
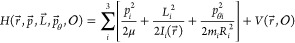
5where  is the reduced mass of the two-particle system, *p*_*i*_ are the canonical momenta of relative molecular motion, *L*_*i*_ are the three components of angular momentum, *I*_*i*_ are the three moments of inertia, *p*_θ*i*_ are the canonical momenta of intermolecular torsional motion, and *m*_*i*_ are the reduced masses of the respective modes. *r*_*i*_ are the components of the intermolecular distance vector *r⃗*, and *R*_*i*_ are the radii of gyration, which are presumably constants.  is the distance and orientation-dependent interaction potential describing the shape of the van der Waals well.

Now, as we are only interested in making an order-of-magnitude estimation, we may further simplify the Hamiltonian into a slightly more manageable form. First, as anisotropic effects on the van der Waals well are difficult to model accurately, they will be ignored for now, reducing  to an isotropic *V*(*r*). Thus, only the magnitude of the relative momentum vector matters: . For the rotational motion, we will be making the assumption that all bimolecular complexes are near-prolate rotors, for which *I*_1_ < *I*_2_ ≈ *I*_3_. We may further approximate *I*_2_ ≈ *I*_3_ ≈ μ*r*^2^, which is increasingly true as the distance between the two molecules increases (see Table S4 and Figure S5 in the Supporting Information for understanding to which extent these approximations are accurate). The rotational axis that *I*_1_ corresponds to passes through the coordination axis of the bimolecular complex (Figure 1), and as such, presumably stays constant even as the intermolecular distance increases. The Hamiltonian is now
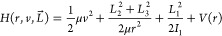
6

Now, we can solve Hamilton’s equations of motion
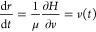
7a
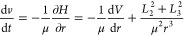
7b

Here, we see that, with these physical assumptions, the angular momentum around the two nonprincipal axes is a constant of motion. We may thus combine them into one constant *L*_2_^2^ + *L*_3_^2^ = *L*_φ_^2^. This is a property of a symmetric rotor, and we may assume that this is approximately the case for a near-symmetric rotor. We also see that the centrifugal effects of rotation around the principal axis do not contribute to the dissociative motion but only to stretching of chemical bonds. Thus, it disappears from the equation of motion, leaving only the centrifugal contribution from the other two rotational axes. These equations of motion can be used to simulate dissociation trajectories at a wide range of initial values for (*v*, *L*_φ_). If the function used for *V*(*r*) is simple enough, this can be performed in rapid succession to cover the space of the most likely dissociating trajectories. Furthermore, with a suitable function of how the escape rate *k*_esc_ depends on the initial relative velocity and angular momentum, one is able to connect the simulated well escape rates to a statistical dissociation rate *k*_d_(*T*)
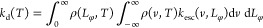
8

where ρ(*x*, *T*) denotes a Boltzmann-distributed probability density. For the velocity distribution ρ(*v*, *T*), we are using the Maxwell–Boltzmann distribution (eq 9). This formulation neglects negative values of *v*, as it is assumed that these will only result in an elastic collision followed by dissociation trajectory identical to the corresponding positive *v* value. The numerical accuracy loss resulting from this neglect should be negligible compared to our overall uncertainty.
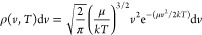
9where *k* is the Boltzmann constant. The usage of probability distributions warrants its own discussion in light of the possible non-ergodicity of bimolecular complexes. Here again, it is convenient that *v* and *L*_φ_ both express the relative motion of the molecules without taking a stand on which molecule the thermal energy is localized in. The bigger problem is that the complex may not be thermalized, in which case the true shape of ρ is uncertain. As the main focus of the model is to make order-of-magnitude estimations, we nevertheless assume a Boltzmann-like distribution where *T* is a parameter that may be higher or lower than the ambient temperature. While being crude, this is not an entirely physically unfounded assumption, as the Boltzmann distribution is the probability distribution that maximizes entropy when ⟨*E*⟩ is constant.^38^ The assumption that random nonequilibrium energy transfers will shift time-dependent probability distributions into Boltzmann-like shapes is already built into the ’exponential down’ energy transfer models typically used in master equation models.^39^

### Probability Distribution of Angular Momentum

ρ(*L*_φ_, *T*) is less straightforward, as there is no closed-form expression for the energy of an asymmetric rotor. It will be derived below. As the impact of angular momentum on the dissociative trajectories is nonlinear, a multilevel approach for its inclusion in the trajectory simulations was implemented

1. Ignore centrifugal acceleration completely by solving eq 7 as if *L*_φ_ = 0.
2. Simulate trajectories only at the average value, ⟨*L*_φ_^2^⟩_*r*=*r*_e__, calculated using the spectroscopic rotational constants of the bimolecular complex at its equilibrium geometry.
3. Vary *L*_φ_ over the full range of reasonably probable angular momenta, and determine *k*_esc_(*v*, *L*_φ_) by surface fit.

The energy levels of an asymmetric rigid rotor by King et al.,^40^ where *A* ≥ *B* ≥ *C* are the spectroscopic rotational constants in energy units, *J* is the rotational quantum number, and *K* is the quantum number for rotation around the principal axis, are given by
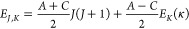
10

where κ is Ray’s asymmetry parameter,^41^ whose span is −1 ≤ κ ≤ 1
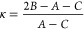
11

As mentioned previously, bimolecular complexes may be approximately treated as near-prolate rotors, for which κ → −1. (rotor type I or ’the limiting prolate spheroid’ by King et al.) In this case, the energy levels may be approximated with
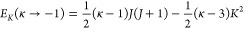
12

Assuming this is approximately accurate, we plug this into eq 10
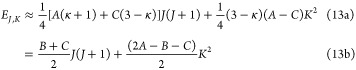
13a

As shown in eq 7, the rotation along the main axis does not contribute to dissociative motion. We can thus neglect the  term, leaving only
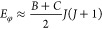
14the crucial detail being that this expression is only accurate in the limit κ → −1, in which *L*_φ_ and *L*_1_ are separable in the equations of motion and rotational energy. For complexes that deviate significantly from this limit, both our trajectory simulations and probability distribution of *L*_φ_ may be inaccurate. Values of κ for our investigated complexes are presented in Table S4 in the Supporting Information. Now, we are ready to start formulating the probability distribution. As we only have two rotational degrees of freedom, the Boltzmann distribution for eq 14 is
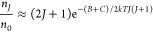
15

As the distances between rotational energy levels are typically quite low, we will approximate the discrete probability distribution as a continuous one. This requires integration over *L*_φ_, which for a molecule-sized system is in the 10^–32^ kgm^2^/s order of magnitude. Tiny floats like these typically cause problems for numerical integrators, and thus we perform a variable change to circular velocity . The transformation from *J* to *l* is
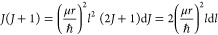
resulting in the normalized probability distribution
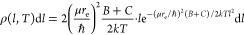
16

Here, it is convenient to lump the constants together: . Now, the average angular momentum to be used in the *l*^2^ = constant simulations is determined by
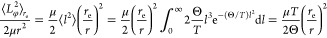
17

Values of ⟨*l*^2^⟩ are listed in Table S5 in the Supporting Information.

Note that the energy expression does not include a centrifugal distortion term, despite the fact that bimolecular complexes quite obviously are nonrigid rotors. This is for mathematical convenience, as subtracting a term proportional to *J*^2^(*J* + 1)^2^ from the energy results in a probability distribution proportional to *l*e^–Θ*l*^2^+Θ_D_*l*^4^^, which is a diverging integral. This can be remedied by correcting the normalization constant with a factor *N*_D_ determined by integrating numerically up to some cutoff value *l*_c_, after which the first-order centrifugal distortion term stops being physically feasible. The centrifugal distortion-corrected distribution, as a whole, is
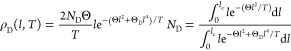
18

Centrifugally corrected probability distributions were not used in the final results, as the impact of the distortion term turned out to be negligible. See the section “Centrifugal Correction” in our Supporting Information for the full details, as well as derivations of *l*_c_ and Θ_D_.

### Reduced 1D Trajectory Simulation

One of the most common models for *V*(*r*) used in models for irreversible dissociation^42^ reactions is the Long-Range Morse Potential,^43^ combining the Morse potential, which qualitatively describes intramolecular interactions at close distance with long-range electrostatic multipole potentials, which qualitatively describe the interaction as distances where no chemical bonding occurs. The problem with using such a scheme in our case is that similar general close-range models do not exist for bimolecular complexes. As such, mathematical simplicity was preferred in the choice of *V*(*r*), based on the logic that the energetics and physical properties of the complex will be more important than the shape of the potential energy surface (PES) for determining the order of magnitude of the dissociation rate, and as such, we may model the shape fairly crudely. Our choice for a go-to model for *V*(*r*) was the Lennard-Jones potential (LJ, eq 19) for two reasons: first, solving the equations of motion (eq 7) with this model is efficient, and second, plugging in literature values for the dissociation energy *D* and the intermolecular equilibrium distance *r*_e_ is exceedingly easy and does not require performing additional costly and cumbersome long-distance PES scans. In reality, the attractive component of *V*(*r*) is often stronger than *r*^–6^, but as seen in the section (In)accuracy of Lennard-Jones Potential of the Supporting Information, this physical inaccuracy does not cause order-of-magnitude errors. If there are multiple potential wells present in the van der Waals well, *D* and *r*_e_ should be chosen based on which well is assumed to be most probably occupied. Occupation probabilities for each distinct well can in principle be determined from the *G* value at the bottom of the well, but these are difficult to calculate accurately for reasons covered in the Introduction section.
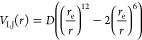
19

A large number of dissociation trajectories were sampled for each bimolecular complex. The well escape rate *k*_esc_ was defined as the inverse of the time it took for the system to reach a predetermined cutoff distance of 3*r*_e_, at which the potential energy is around 0.0027 of the well depth in the LJ potential. For our test set of alkoxyl radical complexes, this corresponds to 9–16 Å depending on the size of the radicals, which is far enough that the orbital overlap between the molecules is likely negligible but close enough that collision between the complex and bath gas molecules are unlikely to impact dissociative trajectories in atmospheric conditions, the mean collision-free path for an N_2_ gas molecule at *p* = 1 atm and *T* = 298.15 K being 91 nm.^44^ In other words, one may assume with reasonable certainty that molecules reaching this distance will not rebound.
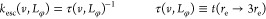
20

As the equations of motion 7 have been reduced to one dimension, the dynamics of the system is simple enough that the Euler integration scheme turned out to be the most efficient integration method, as only one value of *r*(*t*) and *v*(*t*) each needs to be saved between iterations. A timestep of Δ*t* = 5 × 10^–16^ s was used in all trajectory simulations. The resulting τ values corresponded almost exactly to those determined with Δ*t* = 1 × 10^–18^ s in a couple of test runs. The time iteration was thus deemed good enough. Three different codes for simulating a given sample of trajectories were written for each of the three levels of theory presented in the introduction to the Methodology Section.

### Trajectory Sampling

Simulated trajectories were sampled based on simple probabilistic criteria: the probability density ρ(*v*, *l*, *T*) of the sampled parameter pair (*v*, *l*) should be within a factor of 100 of ρ_max_, and the maximum probability density of all dissociative trajectories. These criteria was chosen to ensure that the empirical function *k*_esc_(*v*, *l*) is based on the most likely dissociation pathways. All sampling was performed with the temperature *T* = 298.15 K, and it is worth pointing out that varying the temperature greatly will result in a slightly different set of trajectories, which will have an impact on *k*_esc_(*v*, *l*). Nevertheless, this function ought to be considerably less sensitive to temperature compared to the probability distribution.

In this reduced one-dimensional (1D) framework, trajectories are defined as dissociative if they have enough linear translational energy to overcome the effective (centrifugal-dependent) dissociation energy. In other words, there is a cutoff velocity *v*_c_(*l*), above which trajectories are dissociative and below which they are nondissociative. If centrifugal effects are neglected, this can be determined from the conservation of energy
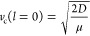
21

**Figure 2:**
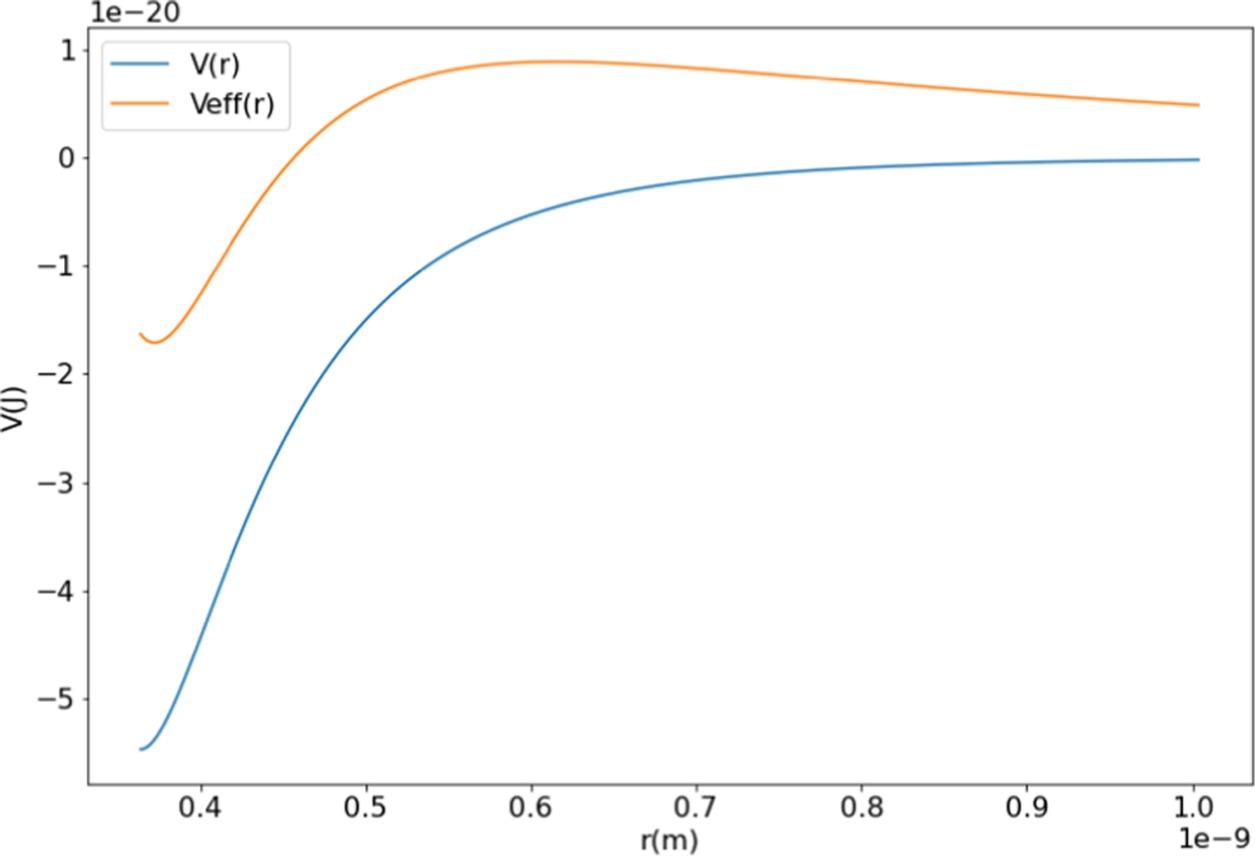
An example curve of *V*_eff_(*r*) compared to *V*(*r*) with the centrifugal barrier present. The variables used are the physical parameters for MetO-ProOHO at *l* = 1451 m/s.

With centrifugal acceleration present, this is slightly more tricky. In principle, this is accomplished by finding the two roots of the effective potential (eq 22, Figure 2; analogous to eq 6 without the translational energy) corresponding to the bottom of the well and the long-range transition state.
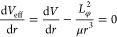
22

If *V*(*r*) = *V*_LJ_(*r*), this expression results in an inconvenient polynomial. Approximate analytical expressions of the two roots can be determined by a Taylor series in the vicinity of *r*_e_ for the bottom of the well and by approximating  for the centrifugal barrier, respectively, but the accuracy of these approximations trails off at higher values of angular momentum. It is therefore more convenient to numerically determine *D*(*l*) = max(*V*_eff_) – min(*V*_eff_) for the range of *l* most relevant for the trajectory sampling and fit the results to a second-order polynomial (eq 23). This is done in the code if one chooses to include centrifugal effects. The values of the α and β coefficients are set to be always positive, as the effective dissociation energy *D*(*l*) should decrease monotonically as a function of *l*. Values of α and β as well as fit *R*^2^-coefficients are presented for our test set in Table S5 in the Supporting Information.
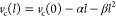
23

Next, finding the conditional maximum ρ_max_. We start by finding the maximum of ρ(*L*_φ_, *T*). By differentiating eqs 9 and 16, we find
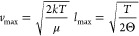
24By comparing eqs 21, 23, and 24, we can now formulate a function for the conditional maximum ρ_max_
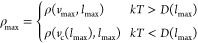
25

Here, it is perhaps worth noting that *kT* < *D*(*l*_max_) applied for all of the complexes studied in this work and is quite possibly true for all systems of chemical interest. Nevertheless, *kT* > *D*(*l*_max_) is still theoretically possible for weakly bound complexes at high temperatures.^1^ As mentioned previously, all values of (*v*,*l*) which met the conditions ρ(*v*,*l*) ≥ 10^–2^ρ_max_, *v* ≥ *v*_c_(*l*) were sampled, using the grid Δ*v* = 1 m/s for levels of theory 1 and 2, and Δ*v* = 5 m/s and Δ*l* = 10 m/s for the *l*-variable simulations.

### Fitting Trajectory Results to a Function

In order to integrate the trajectory results over a probability distribution, as shown in eq 8, we must find an empirical function *k*_esc_(*v*, *l*) that fits the data. All fits were performed using the scipy.optimize python library.

For the *l* = 0 simulations, we assume *l* = 0 and therefore only have to consider the dependence of *k*_esc_ on the initial velocity. A trend observed from the trajectory data was a decay to zero at velocities close to the escape velocity (lim_*v*→*v*_*c*_^+^_*k*_esc_(*v*) = 0) and an approximately linear dependence on *v* at higher velocities, which is a perfectly sensible behavior in terms of conservation of energy. The decaying behavior seems to be probably most accurately described by tanh(*k*(*v* – *v*_c_)) or some other function which by definition is zero at *v* = *v*_c_, but as we want a function that is easy to fit and integrate. Therefore, this decay is modeled with a simple exponential function. As a whole, our empirical model function is
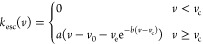
26where, in order to help keep track of the units, we have expressed all parameters in velocity units whenever possible. For the *l*^2^ = constant simulations, the same function was used with minor alterations
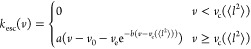
27In the *l*-variable simulations, the treatment of *l* as a variable means that a surface fit is required, increasing the difficulty of finding good parameters. For this reason, the three parameters (*a*, *v*_0_, *d*) determined for the *l* = 0 simulations were inserted as constants into the surface fit to ensure that *k*_esc_(*v*, 0) is compatible with our *k*_esc_(*v*) function. The coefficients (α, β) from eq 23 were similarly included as constants. Thus, the uncertainty of the *l*-variable results is dependent on three fits, namely, eqs 23, 26, and 28. Aside from these five constants, three parameters were added to account for the additional impact of centrifugal effects.
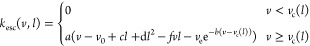
28As these equations are completely empirical, one should note that the values of the parameters will depend on the sampled trajectories. It is therefore important that the function describes the most likely dissociative trajectories well. The fits were evaluated using not only the usual *R*^2^ coefficient but also a Boltzmann-weighted *R*^2^ coefficient. All of the *R*^2^-coefficients of these curve and surface fits are presented for our test set in Table 2.
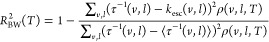
29

### Integration over the Probability Distribution

Here, we will present the integrals in eq 8 one *l*-approach at a time, showing which components are analytically integrable and which must be integrated numerically.

For the *l* = 0 simulations, we combine eqs 26 and 9 and complete  into a square
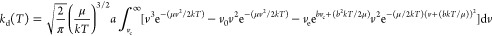
From which, a closed-form solution can be determined using standard Gaussian integrals
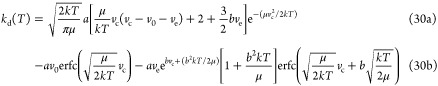
30aFor the *l*^2^ = constant simulations, the equation is the same, but the parameters (*a*, *b*, *v*_0_, *v*_e_, *v*_c_) are different.

For the *l*-variable simulations, we must integrate over *l* as well as *v*. As such, we start with the expression in eq 30 but replace *a* with *a*(1 + *fl*) and *v*_0_ with (*v*_0_ – *cl* – *dl*^2^) in accordance with eq 28. Noting that *v*_c_ is now a variable as per eq 23, we must perform the integral over *l* numerically, as the first term in eq 30 includes a fourth-order polynomial in the exponent, and the other two include a variable inside a complementary error function. Expressed as a whole, the integral is
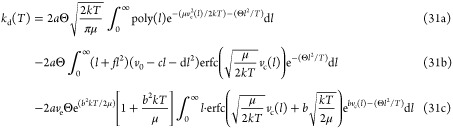
31awhere Poly(*l*) is a sixth-order polynomial with the terms (*v*_c_(0) is denoted as *v*_c_ to avoid clutter)
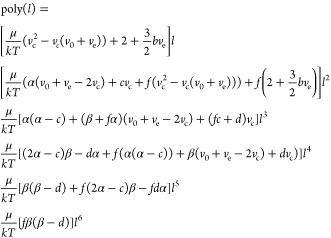


## Results and Discussion

The final canonical dissociation rates for our chosen model systems are presented in Table 1. The trajectory rates are compared to detailed balance rates from Source (30) and to RRKM dissociation rates calculated based on a dissociation energy scaled ωB97XD/jul-cc-pVDZ^45,46^ PES scan in Gaussian 16^47^ starting from the geometries optimized in sources (30) and (35). From these results, we see that the RRKM rates are equally unphysical as the detailed balance rates, meaning we cannot use this to benchmark our results. What we can do instead is refer to experimental results,^48^ according to which the products of dissociation and a H-shift reaction are both observed for the (MetO)_2_ and (EtO)_2_ systems, implying that these must have comparable rates. According to our best estimates (ωB97XD/aug-cc-pVTZ), the H-shift rates for the (MetO)_2_ and (EtO)_2_ complexes are 5.42 × 10^8^ s^–1^ and 1.07 × 10^8^ s^–1^, respectively. Our rates are 1–2 orders of magnitude larger, which in this context is likely good enough, as finding an electronic structure method that accurately models the kinetics of triplet state alkoxyl complexes is still work in process.^32^ All intermediary parameters can be found under the section Complex Parameter Data in the Supporting Information. As seen from the results, the dissociation rate is exponentially dependent on the dissociation energy, as is expected from any process at thermal equilibrium. This is partially because we have neglected most of the more nonlinear effects on the dissociation, such as the coupling of inter- and intramolecular vibrational modes, and the anisotropicity of the van der Waals well. Arguably, the dissociation rates would still show an exponential dependence on the binding energy if these effects were included however, so our results should be a decent first-order approximation. In order to phenomenologically explain the trends seen in the results, we should consider a hypothetical bimolecular complex with zero binding energy. In this case, the escape rate is simply , and the canonical dissociation rate without centrifugal effects is
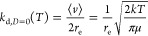
32

**Table 1:** Canonical Rate Coefficients at *T* = 298.15 K (in Unit s^–1^) for the Model Complexes (All in the Triplet State) in All Three Levels of Theory^a^

complex	*D* (kcal/mol)	*l* = 0	*l*^2^ = ⟨*l*^2^⟩	variable *l*	DB^30^	RRKM
(MetO)_2_	3.32	1.45 × 10^10^	3.58 × 10^10^	7.56 × 10^10^	6.21 × 10^13^	9.72 × 10^13^
(EtO)_2_	5.61	2.80 × 10^08^	8.08 × 10^08^	2.57 × 10^09^	1.16 × 10^11^	1.89 × 10^12^
(ProO)_2_	4.70	1.04 × 10^09^	3.18 × 10^09^	8.87 × 10^09^	1.11 × 10^13^	5.20 × 10^13^
(AceO)_2_	6.85	2.99 × 10^07^	1.01 × 10^08^	1.51 × 10^09^	3.97 × 10^11^	2.48 × 10^11^
(ButO)_2_	4.55	1.05 × 10^09^	3.05 × 10^09^	9.03 × 10^09^	9.71 × 10^13^	8.24 × 10^13^
*R*,*R*-(BuOHO)_2_	13.42	5.72 × 10^02^	2.26 × 10^03^	2.96 × 10^04^	7.48 × 10^09^	
*R*,*S*-(BuOHO)_2_	8.02	3.72 × 10^06^	1.34 × 10^07^	8.10 × 10^07^	6.29 × 10^13^	
*R*-alkoxy,*R*-nitroxy-α-pin	10.71	2.50 × 10^04^	1.36 × 10^05^	2.42 × 10^06^		
*R*-alkoxy,*S*-nitroxy-α-pin	10.18	5.73 × 10^04^	2.21 × 10^05^	2.43 × 10^06^		
*S*-alkoxy,*R*-nitroxy-α-pin	18.19	1.07 × 10^–01^	4.84 × 10^–01^	2.67 × 10^01^		
*S*-alkoxy,*S*-nitroxy-α-pin	10.83	2.11 × 10^04^	8.85 × 10^04^	1.45 × 10^06^		
(α-pin-O_3_–RO)_2_	13.682	2.23 × 10^02^	1.01 × 10^03^	3.67 × 10^04^		
MetO-EtO	4.570	1.67 × 10^09^	4.56 × 10^09^	1.20 × 10^10^		
MetO-ProO	3.704	6.25 × 10^09^	1.78 × 10^10^	4.48 × 10^10^		
MetO-AceO	3.336	8.69 × 10^09^	2.48 × 10^10^	5.99 × 10^10^		
MetO-ProOHO	7.872	7.95 × 10^06^	2.99 × 10^07^	2.01 × 10^08^	5.55 × 10^11^	6.69 × 10^10^
MetO-BuOHO	6.993	2.94 × 10^07^	1.25 × 10^08^	1.02 × 10^09^		
EtO-ProO	5.221	5.02 × 10^08^	1.50 × 10^09^	4.67 × 10^09^		
EtO-AceO	5.983	1.48 × 10^08^	4.90 × 10^08^	2.05 × 10^09^		
EtO-ProOHO	9.486	5.08 × 10^05^	1.87 × 10^06^	1.44 × 10^07^		
EtO-BuOHO	8.617	1.86 × 10^06^	7.62 × 10^06^	6.77 × 10^07^		
ProO-AceO	5.835	1.59 × 10^08^	5.01 × 10^08^	1.90 × 10^09^		
ProO-ProOHO	8.980	1.02 × 10^06^	3.62 × 10^06^	2.08 × 10^07^		
ProO-BuOHO	8.326	2.69 × 10^06^	1.01 × 10^07^	5.97 × 10^07^		
AceO-ProOHO	9.591	3.43 × 10^05^	1.23 × 10^06^	7.91 × 10^06^		
AceO-BuOHO	9.898	1.99 × 10^05^	7.64 × 10^05^	5.61 × 10^06^		

a DB = detailed balance. The dissociation energies are presented for comparison, as they are clearly the most important parameters determining the dissociation rate. The number of decimals used for the dissociation energies is kept the same from the original sources. As seen in the last column on the right, dissociation rates calculated assuming detailed balance of association and dissociation overestimate the dissociation rate up to unphysical values. This is pointed out in the original article as well.^30^

This is the effective upper limit for dissociation rates with our methodology. It is between 2 × 10^11^ s^–1^ and 10 × 10^11^s^–1^ for the complexes in our test set, which is on par with the loosest intermolecular vibrations,^49^ translating to 7–33 cm^–1^. The detailed balance model, on the other hand, returns a dissociation rate of 8 × 10^18^ s^–1^ for our hypothetical zero energy complex, assuming an association rate of 10^–10^ molecule/cm^3^·s and a Δ*G* of +22*RT* (see the Supporting Information). Using eq 32 as the basis, the *l* = 0 dissociation rates should be described by the following toy model
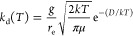
33where *g* is a fudge factor correcting for the nonlinear time-evolution of the relative velocity during the trajectories. Its value is between 4.01 and 10.37 for our test set and depends linearly on *D*. The centrifugally corrected results are more variable, more nonlinear, and also more uncertain due to the near-symmetric approximation used in the determination of the probability distribution. What we do see is that the *L*_φ_^2^ = ⟨*L*_φ_^2^⟩_*r*=*r*_e__ generally underestimates the rates compared to the model where *l* is treated as a variable. This is to be expected, as centrifugal acceleration decreases the effective dissociation energy nonlinearly, which means that high and relatively unlikely values of *L*_φ_^2^ have a disproportionally large impact on dissociation rates. Overall, the impact of centrifugal effects on the dissociation rate mainly scales with *D*. The *l* = 0 and variable *l* dissociation rates for the smallest and floppiest complexes (MetO-RO) only disagree by a factor of 5–7 for the systems without H-bonds and by 25–30 for the systems with H-bonds. The heavier and more inertial α-pinene derived complexes, for which the centrifugal acceleration should intuitively be smaller, show disagreement between the two rates by factors between 42 and 249. This is because the non-centrifugal *v*_c_(0) is far in the tail end of the probability distribution for these systems, meaning that small decreases in *v*_c_(*l*) have a large impact on the overall rate. As a whole, the dependence of the results on the dissociation energy can crudely be estimated with , where η is between 2 and 3 (Table 2).

**Table 2:** *R*^2^-Coefficients for All of the Curve and Surface Fits^a^

	*l* = 0		*l*^2^ = ⟨*l*^2^⟩					
complex	*R*^2^	*R*_BW_^2^	*R*^2^	*R*_BW_^2^	var. *l R*^2^	(*l*-fit) *R*_BW_^2^	var. *l R*^2^	(both fits) *R*_BW_^2^
(MetO)_2_	0.9996	0.9994	0.9995	0.9992	0.9975	0.9957	0.9976	0.9980
(EtO)_2_	0.9996	0.9994	0.9994	0.9992	0.9989	0.9980	0.9967	0.9974
(ProO)_2_	0.9996	0.9995	0.9991	0.9986	0.9987	0.9978	0.9961	0.9972
(AceO)_2_	0.9996	0.9994	0.9993	0.9989	0.9988	0.9988	0.9913	0.9940
(ButO)_2_	0.9995	0.9993	0.9995	0.9993	0.9987	0.9977	0.9950	0.9967
*R*,*R*-(BuOHO)_2_	0.9997	0.9997	0.9991	0.9987	0.9998	0.9996	0.9953	0.9967
*R*,*S*-(BuOHO)_2_	0.9996	0.9995	0.9992	0.9988	0.9995	0.9993	0.9952	0.9970
*R*-alkoxy,*R*-nitroxy-α-pin	0.9996	0.9994	0.9974	0.9958	0.9995	0.9994	0.9902	0.9938
*R*-alkoxy,*S*-nitroxy-α-pin	0.9997	0.9996	0.9996	0.9994	0.9997	0.9995	0.9943	0.9968
*S*-alkoxy,*R*-nitroxy-α-pin	0.9998	0.9997	0.9993	0.9991	0.9998	0.9997	0.9930	0.9939
*S*-alkoxy,*S*-nitroxy-α-pin	0.9997	0.9996	0.9996	0.9994	0.9996	0.9995	0.9928	0.9957
(α-pin-O_3_–RO)_2_	0.9997	0.9997	0.9995	0.9993	0.9997	0.9996	0.9914	0.9937
MetO-EtO	0.9996	0.9994	0.9994	0.9990	0.9987	0.9975	0.9966	0.9975
MetO-ProO	0.9996	0.9994	0.9994	0.9992	0.9979	0.9965	0.9963	0.9970
MetO-AceO	0.9996	0.9994	0.9996	0.9994	0.9974	0.9957	0.9943	0.9961
MetO-ProOHO	0.9996	0.9995	0.9992	0.9989	0.9993	0.9989	0.9950	0.9962
MetO-BuOHO	0.9996	0.9994	0.9994	0.9991	0.9990	0.9989	0.9932	0.9953
EtO-ProO	0.9996	0.9994	0.9993	0.9990	0.9989	0.9980	0.9962	0.9972
EtO-AceO	0.9996	0.9994	0.9994	0.9992	0.9990	0.9983	0.9956	0.9967
EtO-ProOHO	0.9997	0.9995	0.9992	0.9989	0.9995	0.9992	0.9950	0.9964
EtO-BuOHO	0.9996	0.9995	0.9991	0.9987	0.9994	0.9991	0.9938	0.9957
ProO-AceO	0.9996	0.9994	0.9994	0.9992	0.9991	0.9985	0.9957	0.9970
ProO-ProOHO	0.9996	0.9994	0.9989	0.9983	0.9995	0.9992	0.9958	0.9970
ProO-BuOHO	0.9996	0.9995	0.9987	0.9980	0.9995	0.9991	0.9953	0.9967
AceO-ProOHO	0.9996	0.9995	0.9992	0.9988	0.9996	0.9993	0.9959	0.9972
AceO-BuOHO	0.9996	0.9995	0.9988	0.9982	0.9996	0.9993	0.9952	0.9967

a As you see, the agreement between the function and the trajectory data is in general very good.

One noteworthy thing about the model as a whole is that, as the most impactful parameter for determining the rate, the dissociation energy must be found from the literature or calculated separately. This of course means that the accuracy of the calculated rates is highly dependent on the accuracy of the used value for *D*. By differentiating , we find that, even when not factoring in the nonlinear binding energy-related effects mentioned above, the uncertainty in the rate depends on the uncertainty in *D* by at least , which translates to roughly 1.7*k*_d_ at *T* = 298.15 K if Δ*D* = ±1 kcal/mol. In this context, the neglect of potential surface anisotropy, rovibrational coupling, etc., is unlikely to have a noticeable impact on the uncertainty in rates unless the binding energy is known with high precision.

## Conclusions

In this work, we have suggested a simple and computationally cheap model for calculating dissociation rates for weakly bound bimolecular complexes. Individual molecules are treated as rigid bodies, and this means that several compromises are made in the modeling of the precise dynamics of the complex, such as neglecting the coupling of intramolecular and intermolecular modes. Nevertheless, the model produces physically consistent results that are usable in order-of-magnitude estimations of irreversible dissociation rates in an atmospheric context, or in other situations with sufficiently low gas density for which probability densities can be similarly estimated. This is especially useful for complexes where accurately calculating Δ*G* of complex formation is considerably harder than accurately calculating Δ*E*, or where the typical detailed balance approach fails to determine dissociation rates for other reasons.

## Data Availability

The code (Python 3 for numerical calculations, Bash for decision trees), the output files of the code, and the quantum chemical output files from the PES scans are available in a separate zip directory.

## Supporting Information Available

Derivation of centrifugal corrections to the probability distribution; variation of the trajectory simulation cutoff distance; test calculations related to the (In)accuracy of the Lennard-Jones potential; notes of how to account for PES anisotropy; values for all physical and derived model parameters for the test set; visualizations of *v*,*l* probability distributions; xyz-geometries used for determination of equilibrium distance parameter *r*_e_; an additional discussion on the non-ergodicity of bimolecular complexes; output files of the quantum chemical ωB97XD/jul-cc-pVDZ PES scans performed for the (In)accuracy of the Lennard-Jones potential section (PDF)
Complex parameter data (ZIP)

## Footnotes

1 Then again, dissociation likely outcompetes everything if this is the case!
