# Supplementary material for: Simple Physical Model for the Estimation of Irreversible Dissociation Rates for Bimolecular Complexes
Source: J Phys Chem A. 2023 Jul 7;127(28):5956–66. doi: 10.1021/acs.jpca.3c01890 (PMC10364079; doi:10.1021/acs.jpca.3c01890)
Supplement: Supplementary file 1 — jp3c01890_si_001.pdf [file jp3c01890_si_001.pdf]

Supporting Information: Simple physical model for estimation of irreversible dissociation rates for bimolecular complexes

Lauri Franzon*

Department of Chemistry, University of Helsinki,

P.O. Box 55 (A.I. Virtasen aukio 1), 00014 Helsinki, Finland

E-mail: lauri.franzon@helsinki.fi

Centrifugal Correction to Probability Distribution

The centrifugal distortion of rotational energy levels is a well-known effect in spectroscopy, in which we may express the (first-order) distortion as a perturbative correction to the rigid rotor energy (Equation 14 in the main article):

$$E_\varphi \approx \frac{B+C}{2} J(J+1) - F J^2 (J+1)^2 \tag{1}$$

In this section we investigate if centrifugal distortion has any impact of the probability distribution of angular momentum, by deriving the centrifugal distortion constant F for a Lennard-Jones potential well as well as a a suitable cut-off value l_c for the probability distribution. Bimolecular complexes are obviously non-rigid rotors, otherwise they would not be able to dissociate in the first place. Since the Lennard-Jones expression we use for the interaction energy of bimolecular complexes is admittable crude, we will first derive the first-order centrifugal distortion term using a general potential energy before plugging in our Lennard-Jones approximation into it.

First, we have to solve the minimum of the effective potential $V_{eff}(r) = V(r) + \frac{L_\varphi^2}{2\mu r^2}$ as a function of L_φ. We will call this minima r_L. Assuming $r_e \approx r_L$ at all physically reasonable values of L_φ, we take the Taylor series of $V_{eff}(r)$ in the vicinity of r_e up to the second order:

$$\begin{aligned} V_{eff}(r) &\approx V_{eff}(r_e) + \left(\frac{\mathrm{d}V_{eff}}{\mathrm{d}r}\right)_{r_e} (r - r_e) + \frac{1}{2}\left(\frac{\mathrm{d}^2 V_{eff}}{\mathrm{d}r^2}\right)_{r_e} (r - r_e)^2 \\ &= V(r_e) + \frac{L_\varphi^2}{2\mu r_e^2} - \frac{L_\varphi^2}{\mu r_e^3}(r - r_e) + \left[\frac{1}{2}\left(\frac{\mathrm{d}^2 V}{\mathrm{d}r^2}\right)_{r_e} + \frac{3L_\varphi^2}{2\mu r_e^4}\right](r - r_e)^2 \end{aligned}$$

The derivative of the Taylor series is zero if:

$$\frac{L_\varphi^2}{\mu r_e^3} = 2\,(r - r_e)\left[\frac{1}{2}\left(\frac{\mathrm{d}^2 V}{\mathrm{d}r^2}\right)_{r_e} + \frac{3L_\varphi^2}{2\mu r_e^4}\right] \implies r_L = r_e + \frac{r_e L_\varphi^2}{3L_\varphi^2 + \mu r_e^4 \left(\frac{\mathrm{d}^2 V}{\mathrm{d}r^2}\right)_{r_e}} \tag{2}$$

Comparison of this approximation with numerically determined values of r_L for the floppier (MetO-XO) complexes shows that the approximation slightly underestimates the deviation from r_e starting from L_φ values corresponding the final 5-10 percentiles of $\rho(L_\varphi, T)$ at room temperature. For the stronger binding complexes (α-pin-derived) the approximation is accurate for all modelled values of L_φ. As higher values of L_φ contribute more to dissociation rates, the third order Taylor series term might be important for some systems. However, the resulting expression for r_L is a complicated quadratic equation solution, which we will not consider here. Now, let's see what corrections result from applying our Lennard-Jones potential:

$$V_{LJ}(r) = D\left(\left(\frac{r_e}{r}\right)^{12} - 2\left(\frac{r_e}{r}\right)^{6}\right) = \frac{D\left(r_e^{12} - 2r_e^6 r^6\right)}{r^{12}} \tag{3}$$

Plugging in $\left(\frac{\mathrm{d}^2 V}{\mathrm{d}r^2}\right)_{r_e} = \frac{72D}{r_e^2}$ into equation 2 results in:

$$r_L = r_e\left(1 + \frac{L_\varphi^2}{3L_\varphi^2 + 72\mu r_e^2 D}\right) = r_e\frac{4L_\varphi^2 + 72\mu r_e^2 D}{3L_\varphi^2 + 72\mu r_e^2 D} \equiv r_e f(L_\varphi) \tag{4}$$

Next, we want to expand $V_{eff}(r_L) = V(r_L) + \frac{L_\varphi^2}{2\mu r_L^2}$ into a power series in L_φ^{2n}, where $n = 2$ is the first centrifugal distortion term. We again make a Taylor series, this time in the vicinity of $L_\varphi^2 = 0$. Using the partial derivation $\left(\frac{\partial f}{\partial L_\varphi^2}\right)_0 = \left(\frac{\partial f}{\partial r}\right)_{r_e}\left(\frac{\partial r_L}{\partial L_\varphi^2}\right)_0$:

$$V_{eff}(r_L) \approx -D + \frac{L_\varphi^2}{2\mu r_e^2} + \left[\left(\frac{\mathrm{d}V}{\mathrm{d}r}\right)_{r_e}\left(\frac{\partial r_L}{\partial L_\varphi^2}\right)_0 - \frac{L_\varphi^2}{\mu r_e^3}\left(\frac{\partial r_L}{\partial L_\varphi^2}\right)_0\right] L_\varphi^2 + \dots \tag{5}$$

We are able to use the fact that $\left(\frac{\mathrm{d}V}{\mathrm{d}r}\right)_{r_e} = 0$ to our advantage. Differentiating equation 4 with L_φ^2 results in:

$$\left(\frac{\partial r_L}{\partial L_\varphi^2}\right)_0 = \left(\frac{72\mu r_e^3 D}{\left(72\mu r_e^2 D + 3L_\varphi^2\right)^2}\right)_0 = \frac{r_e}{72\mu r_e^2 D}$$

Resulting in a first-order centrifugal correction to the rotational energy of:

$$V_{eff}(r_L) - V_{eff}(r_e) \approx \frac{L_\varphi^2}{2\mu r_e^2}\left[1 - \frac{L_\varphi^2}{72\mu r_e^2 D}\right] = \frac{L_\varphi^2}{2\mu r_e^2} - \frac{L_\varphi^4}{144\mu^2 r_e^4 D} \tag{6}$$

From this, we can derive the 1st order centrifugal coefficient Θ_D (for a Lennard-Jones well) in Equation 18 in the main article:

$$\frac{L_\varphi^2}{2\mu r_e^2} - \left(\frac{L_\varphi^2}{2\mu r_e^2}\right)^2 \frac{1}{36D} = k\Theta l^2 - k\Theta_D l^4 \implies \Theta_D = \frac{k\Theta^2}{36D}$$

As mentioned in the main text, in one includes a first order centrifugal distortion into the rotational energy, the probability distribution $\rho(L_\varphi, T)$ becomes divergent, and we thus need an integration limit below infinity. We call this quantity the critical angular momentum L_c, named after the critical temperature because we are deriving it using the same method as critical temperatures and pressures are derived from the Van der Waals gas law.[S1] We look for an inflection point in $V_{eff}(r)$ at which both the first and second derivatives are zero:

$$\left(\frac{\mathrm{d}V_{eff}}{\mathrm{d}r}\right)_L = \left(\frac{\mathrm{d}^2 V_{eff}}{\mathrm{d}r^2}\right)_L = 0 = \frac{\mathrm{d}V}{\mathrm{d}r} - \frac{L_\varphi^2}{\mu r^3} = \frac{\mathrm{d}^2 V}{\mathrm{d}r^2} + \frac{3L_\varphi^2}{\mu r^4}$$

This equality of these two functions should only apply at a specific value of L_φ. Thus we find the critical angular momentum at the intersection of the following two functions of r:

$$L_c^2 = \mu r^3 \frac{\mathrm{d}V}{\mathrm{d}r} = -\frac{\mu r^4}{3}\frac{\mathrm{d}^2 V}{\mathrm{d}r^2} \tag{7}$$

For a Lennard-Jones well:

$$12D\mu\left[\frac{r_e^6}{r_c^4} - \frac{r_e^{12}}{r_c^{10}}\right] = 4D\mu\left[7\frac{r_e^6}{r_c^4} - 13\frac{r_e^{12}}{r_c^{10}}\right] \implies \frac{r_e^{12}}{r_c^{10}} = \frac{2}{5}\frac{r_e^6}{r_c^4} \implies r_c = \sqrt[6]{\frac{5}{2}}r_e$$

$$L_c^2 = 12D\mu r_e^2\left[\left(\frac{2}{5}\right)^{\frac{2}{3}} - \left(\frac{2}{5}\right)^{\frac{5}{3}}\right] \implies L_c \approx 1.977\sqrt{D\mu}r_e \implies l_c \approx 1.977\sqrt{\frac{D}{\mu}}$$

Values for all centrifugal distortion-related parameters are found in Table S6. A visualisation of the difference between $\rho_D(l, T)$ (Equation 18 in the main article) and the centrifugally uncorrected $\rho(l, T)$ is shown in Figure S1, based on which it was judged that centrifugal distortion corrections will not impact the average dissociation rates significantly.

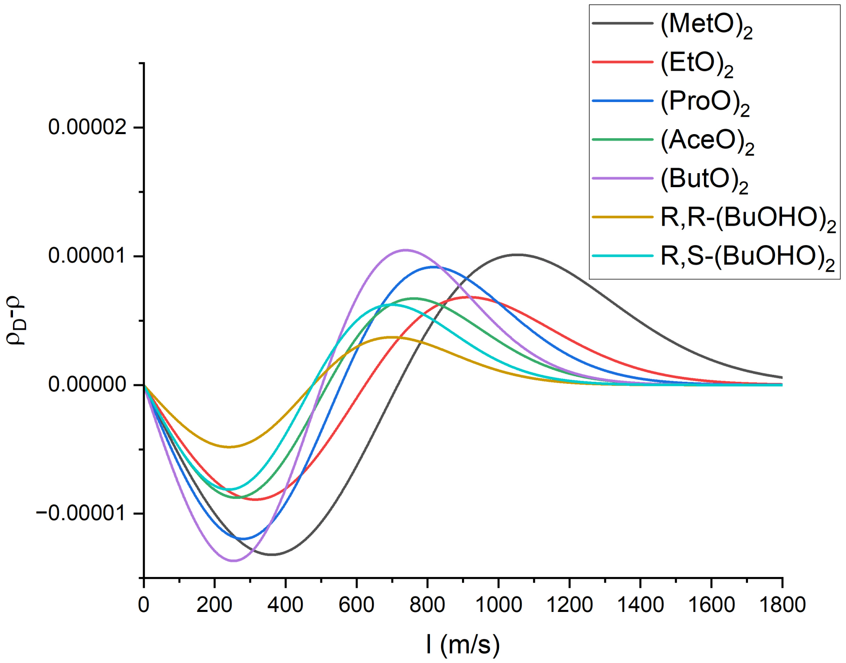

Figure S1: The difference between the corrected and uncorrected probability distributions of angular momentum. All observed changes are less than 10^{-3} of the peak value of the respective uncorrected distribution (0.00214 $\frac{\mathrm{s}}{\mathrm{m}}$ for $(\mathrm{MetO})_2$.)

Variation of Cutoff Distance

Trajectories were simulated at the $l = 0$ level of theory for a smaller sample of complexes to test the sensitivity of the results to the chosen cutoff distance. The results are seen in Table S1. The dependence of the dissociation rate on the cutoff distance r_c should eventually converges to $k_d(T) \propto r_c^{-1}$ one r_c is far enough, but this seems to not be quite true yet at cutoff distance 20-30 Å. A noticeable trend in these results is a slower convergence to the stated dependence for the complexes with further equilibrium distances r_e, which is consistent with

the fact that the potential gradient is higher closer to equilibrium (Figure S2). The trends seen here are the reason why the cutoff distance is set to depend on r_e in the trajectory simulation code, as this means that the ratio $V(r_c)/D$ will always be the same (≈ -0.0027) at the cutoff for each trajectory.

Table S1: Dissociation rates at various cutoff distances.

Complex	10 Å	15 Å	20 Å	30 Å	k_d (20 Å) / k_d (30 Å)
$(MetO)_2$	$1.39{\cdot}10^{10}$	$7.75{\cdot}10^{09}$	$5.32{\cdot}10^{09}$	$3.25{\cdot}10^{09}$	1.63
$(EtO)_2$	$3.59{\cdot}10^{08}$	$1.90{\cdot}10^{08}$	$1.27{\cdot}10^{08}$	$7.59{\cdot}10^{07}$	1.68
$(ButO)_2$	$1.66{\cdot}10^{09}$	$8.47{\cdot}10^{08}$	$5.62{\cdot}10^{08}$	$3.32{\cdot}10^{08}$	1.70
R,R-$(BuOHO)_2$	$8.74{\cdot}10^{02}$	$4.14{\cdot}10^{02}$	$2.69{\cdot}10^{02}$	$1.56{\cdot}10^{02}$	1.73
R,S-$(BuOHO)_2$	$5.99{\cdot}10^{06}$	$2.93{\cdot}10^{06}$	$1.93{\cdot}10^{06}$	$1.11{\cdot}10^{06}$	1.73
S-alkoxy,R-nitroxy-α-pin	$3.92{\cdot}10^{-01}$	$1.27{\cdot}10^{-01}$	$7.62{\cdot}10^{-02}$	$4.17{\cdot}10^{-02}$	1.83
S-alkoxy,S-nitroxy-α-pin	$5.25{\cdot}10^{04}$	$2.12{\cdot}10^{04}$	$1.33{\cdot}10^{04}$	$7.51{\cdot}10^{03}$	1.78

(In)accuracy of Lennard-Jones Potential

$$V(r) = D\left[\left(\frac{r_e}{r}\right)^{12} - 2\left(\frac{r_e}{r}\right)^{6}\right] \tag{8}$$

As covered in the main article, the Lennard-Jones potential (Equation 8) was used to model the total interaction potential for purposes of simplicity rather than accuracy. The main assumption behind this choice was that the depth of the potential well has a much larger impact on the overall dissociation rate than the curvature of the interaction potential at distances $r > r_e$. In this section we will interrogate that assumption. Many popular force fields in Molecular Dynamics make use of the electric field multipole expansion for intermolecular interactions, resulting in a function with the general shape 9. We therefore find it noteworthy that this model is accurate for long-range interactions only, at which the intermolecular distance significantly exceed the intramolecular distances.[S21] In other words, this is not a good model for close-range intermolecular interactions. Unfortunately, as seen in the typical interaction potential curve sketched in Figure S2, the close-range interactions

[1] The cited source discusses this in terms of systems composed of multiple classical charges, but in our case the extent of orbital overlap is a better measure for when the long-range regime starts being a good model for intermolecular interactions. This discussed briefly in source[S3]

of the molecules are also the most important for determining the relative velocity of the molecules along the dissociative trajectory, as that is where the $\nabla V(r)$ is the largest. This means that explicit quantum chemical calculations varying the intermolecular distance r are required to determine $V(r)$ with reasonable accuracy.

$$V(r) = \sum_{n=1}^{\infty} \frac{\pm V_n}{r^n} \tag{9}$$

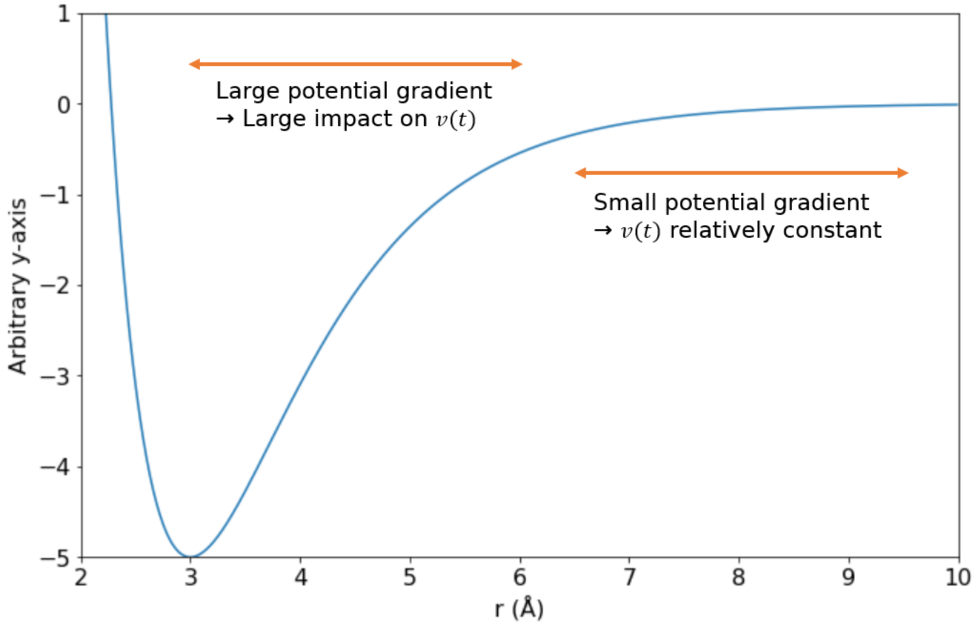

Figure S2: A visualisation of why long-range approximations of the potential energy are not accurate for calculating dissociation.

The accuracy of the Lennard-Jones function by performing quantum chemical potential energy scans on three reasonably small bimolecular complexes with different intermolecular bonding properties: $(MetO)_2$, $(AceO)_2$ and MetO-ProOHO. The scans were performed in Gaussian 16[S4] as a series of constrained geometry optimizations with the two radicals at different intermolecular distances generated from the global minimum geometry provided in the original source. The distance between the two centers of mass was calculated for all the optimized geometries. The electronic structure method chosen for the geometry optimization was ωB97X-D/jul-cc-pVDZ,[S5,S6] which is one 'month' and one basis function step down

from the basis set used in reference S11. The resulting (electronic) energies were compared to those calculated for the optimized bimolecular complex and the isolated radicals using the same level of theory, and the results are presented in Figure S3. As seen in the figure, The attractive interaction is clearly stronger than r^{-6} for $(\mathrm{MetO})_2$ and $(\mathrm{AceO})_2$, whereas for MetO-ProOHO it is somewhat weaker. This is somewhat counterintuitive, as the latter is a H-bonded complex, but a closer look at the data points above the LJ curve showed that the ProOHO molecule prefers prefers an intramolecular H-bonding structure at these distances, explaining the sharper decline in binding energy as a function of r.

For each of the three complexes, a simple trial function that approximates the $V(r)$ data better than the Lennard-Jones potential was chosen. Dissociation trajectories were simulated on levels of theory 1 & 3 and dissociation rates were calculated. The used trial functions are presented in Equation 10, and compared to scan data in Figure S4. The dissociation rates calculated based on the trial functions are presented in Table S2. As seen in the table, the differences are well within the margin of error, justifying the use of the LJ potential for all complexes.

$$V_{(\mathrm{MetO})_2}(r) = D\left[\frac{4}{5}\left(\frac{r_e}{r}\right)^9 - \frac{9}{5}\left(\frac{r_e}{r}\right)^4\right] \tag{10a}$$

$$V_{(\mathrm{AceO})_2}(r) = D\left[\left(\frac{2}{3}\frac{r_e}{r}\right)^{10} - \frac{5}{3}\left(\frac{r_e}{r}\right)^4\right] \tag{10b}$$

$$V_{\mathrm{MetO-ProOHO}}(r) = D\left[\left(\frac{r_e}{r}\right)^{12} - \left(\frac{r_e}{r}\right)^5\right] + D\left(\frac{2}{1+\mathrm{e}^{-14\left(\frac{r}{r_e}-1\right)}} - 2\right) \tag{10c}$$

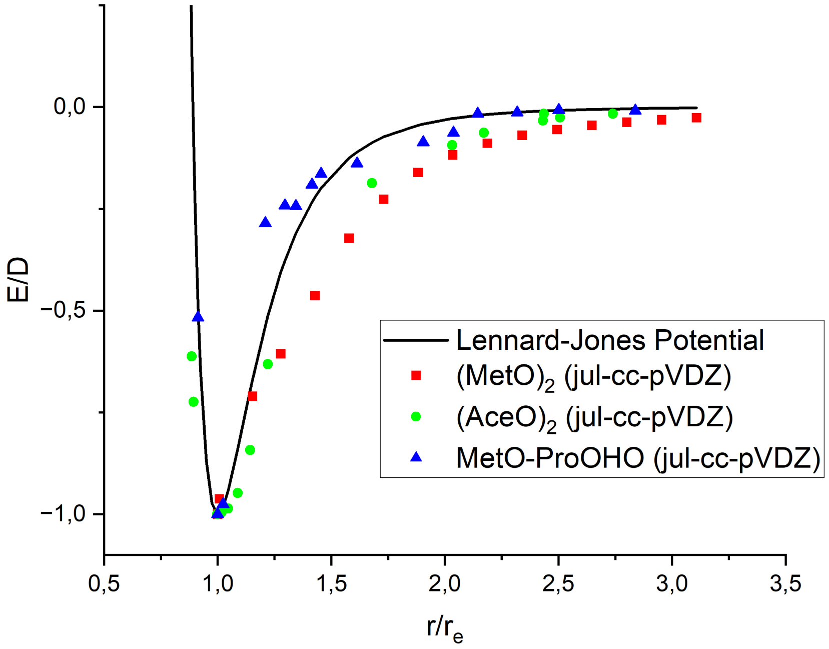

Figure S3: Potential energy curve comparison between the quantum chemical scan results and the Lennard-Jones potential. Note that both axes are presented in dimensionless units to emphasize the shape of the energy curves.

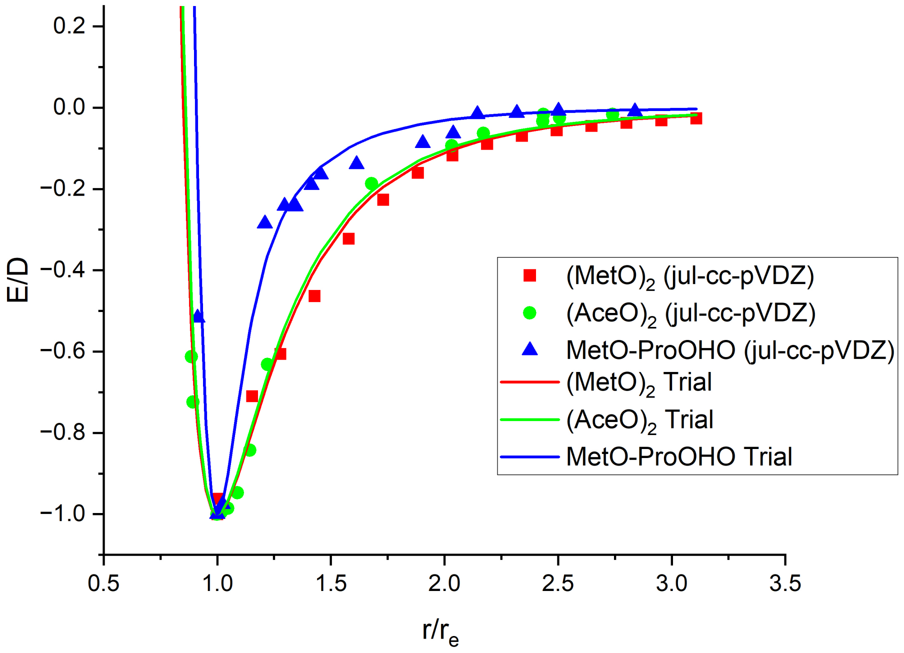

Figure S4: Potential energy curve comparison between the quantum chemical scan results and trial functions presented in Equation 10

Table S2: Comparison of dissociation rates calculated using the Lennard-Jones potential and the trial potentials.

Complex	LJ $V(r)$ $l = 0$	Variable l	Trial $V(r)$ $l = 0$	Variable l
$(\text{MetO})_2$	$1.45{\cdot}10^{10}$	$7.56{\cdot}10^{10}$	$1.47{\cdot}10^{10}$	$8.22{\cdot}10^{10}$
$(\text{AceO})_2$	$2.99{\cdot}10^{07}$	$5.36{\cdot}10^{08}$	$3.13{\cdot}10^{07}$	$7.49{\cdot}10^{08}$
MetO-ProOHO	$7.95{\cdot}10^{06}$	$2.01{\cdot}10^{08}$	$7.63{\cdot}10^{06}$	$1.55{\cdot}10^{08}$

Accounting for potential surface anisotropy

This is a highly tricky aspect to consider in the trajectory simulations. We will therefore not present results on the subject, only suggest a protocol for tackling it with the given tools. Firstly, we assume the orientation-dependent interaction potential $V(\vec{r}, \mathcal{O})$ is separable into an isotropic component $V(r)$ and a dimensionless 'anisotropy factor' $f(\mathcal{O})$[2].

First we must address the most common method of modelling long-range potential surface anisotropy in physical chemistry, namely using the dipole and quadrupole moments of the molecules to quantify the orientation dependence of $V(r, \mathcal{O})$, because it is unfortunately inadequate for our purpose. There are two reasons for this: Firstly, the usage of multipole moments determined for independent molecules at equilibrium geometry implicitly assumes that geometry stays rigid. In van der Waals complexes, the intermolecular interactions perturb both molecular structures from their respective independent equilibrium geometries, meaning that the multipole moments determined for that geometry is no longer accurate. Secondly, the concept of a dipole moment is only physically well defined at distances noticeably longer than the dipole's characteristic length.[S2] This is most certainly not the case in van der Waals complexes. In other words, one must apply a model that fully accounts for the fact that the molecules are polarizable diffuse charge distributions.

A better close-range anisotropy model would be to consider consider the three intermolec-

[2] This assumption may not be applicable if one of the molecules has significant orientation dependence in polarizability, resulting in $V(r)$ decaying differently depending on the orientation.

ular DOF which couple into free rotational modes at high separation, that is, the torsional vibrations. Assuming these are independent, we can most simply model these as three rigid hindered rotors. With that model, the potential energy at equilibrium distance is:[S7]

$$V(r_e, \mathcal{O}) = V(r_e, 0) + \sum_i^3 \frac{U_i}{2}\left(1 - \cos(\sigma_i \theta_i)\right) \tag{11}$$

Where U_i are the hindered rotor barrier heights. As these are intermolecular torsional modes, σ_i is determined by the rotational symmetry of the molecules. Note also that one of the three modes must correspond to the 'altitude coordinate', spanning onlt $0 \leq \theta_3 \leq \pi$ rather than $0 \leq \theta_{1,2} \leq 2\pi$.[S8] According to our simplified Lennard-Jones model (Equation **??**) $V_{LJ}(r_e) = -D$. We may thus reconcile these two models by plugging Equation 11 into D:

$$V(r, \mathcal{O}) = -f(\mathcal{O})\left(\left(\frac{r_e}{r}\right)^{12} - 2\left(\frac{r_e}{r}\right)^{6}\right) \tag{12a}$$

$$f(\mathcal{O}) = D - \frac{U_1 + U_2 + U_3}{2} + \sum_i^3 \frac{U_i}{2}\cos\left(\sigma_i \theta_i\right) \tag{12b}$$

Were we have placed the $U_1 + U_2 + U_3$ expression outside the sum to emphasize that it is a constant. The radial equation of motion changes to:

$$\frac{\mathrm{d}v}{\mathrm{d}t} = \frac{12}{\mu} f(\mathcal{O})\left(\frac{r_e^{12}}{r^{13}} - \frac{r_e^{6}}{r^{7}}\right) + \frac{L_\varphi^2}{\mu^2 r^3} \tag{13}$$

And, if we neglect the quantization of energy levels, we can formulate classical equations of motion for the torsional DOFs as well:

$$\frac{\mathrm{d}\theta_i}{\mathrm{d}t} = \frac{p_{\theta i}}{m_i R_i^3} \qquad \frac{\mathrm{d}p_{\theta_i}}{\mathrm{d}t} = \frac{\sigma_i U_i}{2R_i}\sin\left(\sigma_i \theta_i\right)\left(\left(\frac{r_e}{r}\right)^{12} - 2\left(\frac{r_e}{r}\right)^{6}\right) \tag{14}$$

By assuming that the initial conditions are Boltzmann-distributed, we may derive a canonical probability distribution of (initial values of) the torsional positions θ_i:

$$\int \mathrm{e}^{-\frac{V(r_e,\mathcal{O})+D}{kT}}\mathrm{d}\mathcal{O} = \int_0^{\frac{\pi}{\sigma_3}} \int_0^{\frac{2\pi}{\sigma_2}} \int_0^{\frac{2\pi}{\sigma_1}} \sin\theta_3 \prod_i^3 \mathrm{e}^{-\frac{U_i}{2kT}} \mathrm{e}^{\frac{U_i}{2kT}\cos(\sigma_i\theta_i)}\sigma_i \mathrm{d}^3\theta_i \tag{15}$$

For the first two degrees of freedom, the normalization constant is a modified Bessel function of 0th order:

$$\rho(\theta_{1,2}, T) = \frac{\sigma_{1,2}}{2\pi} I_0\left(\frac{U_{1,2}}{2kT}\right)^{-1} \mathrm{e}^{\frac{U_{1,2}}{2kT}\cos(\sigma_{1,2}\theta_{1,2})}\sigma_{1,2}\mathrm{d}\theta_{1,2} \tag{16}$$

For the third torsional coordinate the normalization constant is a hyperbolic cosecant function:

$$\rho(\theta_3, T) = \frac{U_3}{4kT}\mathrm{csch}\left(\frac{U_3}{2kT}\right) \mathrm{e}^{\frac{U_3}{2kT}\cos(\sigma_3\theta_3)}\sigma_3\mathrm{d}\theta_3 \tag{17}$$

From this point onward, one samples different initial values for the three torsional coordinates similarly as we have done for v and l. As seen from the equations of motion, the torsional coordinates are interdependent with the distance coordinate r, which suggests that the anisotropic effects of the dissociation are nonlinear and hard to quantify. The timestep of the simulation might have to be shortened to ensure that energy is conserved during the simulation. Another added difficulty arising from this approach is that the torsional degrees of freedom have to be characterized and analysed in the first place, and most Hindered Rotor characterization codes are better equipped for finding rotations of covalent bonds.

ΔG for a complex with zero binding energy

For a reaction with net production of gas molecules, the enthalpy change is $\Delta H = \Delta E + RT\Delta n$ in constant temperature. As such the dimerization Gibbs free energy for a hypothetical complex with zero binding energy is:

$$\Delta G = \Delta E - RT - T\left(S_{12} - S_1 - S_2\right) \tag{18}$$

In general E is a sum of the zero-point-corrected potential energy E_0 and the thermal energy E_T. However, with zero binding energy ΔE_0 is zero, and ΔE comes exclusively from the thermal component. Here the partition function-based definition of thermal energy and entropy is useful:

$$E_T - TS = RT^2\frac{\partial \ln Q}{\partial T} - RT\left[1 + \ln Q + T\frac{\partial \ln Q}{\partial T}\right] = -RT(1 + \ln Q) \tag{19}$$

With no binding energy, the six intermolecular modes are all unbounded, and are most accurately described as either translations or rotations. Thus the components of the partition functions are described by:

$$Q_e = \omega \tag{20}$$

$$Q_t = \left(\frac{2\pi mkT}{h^2}\right)^{\frac{3}{2}}\frac{kT}{p} \tag{21}$$

$$Q_r(\mathrm{RR}) = \frac{1}{\sigma}\sqrt{\frac{\pi T^3}{\Theta_A\Theta_B\Theta_C}} = \frac{1}{\sigma\hbar^3}\sqrt{8\pi k^3T^3I_AI_BI_C} \tag{22}$$

where RR refers to the rigid rotor approximation. For the translational modes we use note that we can use the formula for reduced mass to our advantage, as $\frac{m_1^{\frac{3}{2}}m_2^{\frac{3}{2}}}{(m_1+m_2)^{\frac{3}{2}}} = \left(\frac{m_1m_2}{m_1+m_2}\right)^{\frac{3}{2}}$:

$$\frac{Q_t(m_1+m_2)}{Q_t(m_1)Q_t(m_2)} = Q_t\left(\frac{m_1m_2}{m_1+m_2}\right)^{-1} \implies \frac{Q_{t12}}{Q_{t1}Q_{t2}} = \frac{1}{Q_{t,\mu}}$$

Using the reduced mass of a $(\mathrm{MetO})_2$ complex, $Q_{t,\mu}^{-1} = 4.165 \cdot 10^{-7}$ when $p = 1$ atm and $T = 298.15$ K. For the rotational partition function we make two admittedly contradictory assumptions for simplicity: We assume that the rotational symmetry number is $\sigma = 1$ and that both molecules are uniformly dense ($\rho(r, \theta, \varphi) = \rho_0$) spheres of radius $\frac{r_e}{2}$ and total mass $m = \rho_0 V$, and as such all three moments of inertia are:

$$I_{1,2} = 4\pi \int_0^{\frac{r_e}{2}} \rho_0 r^4 \mathrm{d}r = 4\pi\rho_0 \frac{1}{5}\left(\frac{r_e}{2}\right)^5 = \rho_0 \cdot \frac{4\pi}{3}\left(\frac{r_e}{2}\right)^3 \cdot \frac{3}{5}\left(\frac{r_e}{2}\right)^2 = \frac{3mr_e^2}{20}$$

The 'bispherical complex' is this case is a fully symmetric prolate rotor, for which the lower moment of inertia is $I_{12,A} = \frac{3mr_e^2}{20}$, and the two higher are $I_{12,B} = I_{12,C} = \mu r_e^2 = \frac{mr_e^2}{2}$. The quotient of rotational partition functions is:

$$\frac{Q_{r12}}{Q_{r1}Q_{r2}} = \frac{\hbar^3}{\sqrt{8\pi k^3 T^3}} \cdot \left(\frac{3mr_e^2}{20}\right)^{-5} \cdot \left(\frac{mr_e^2}{2}\right)^2 = \frac{800000\hbar^3}{243\sqrt{8\pi k^3 T^3} m^3 r_e^6}$$

Which, using the mass of a MetO radical and the equilibrium distance of a $(\mathrm{MetO})_2$ complex is $\frac{Q_{r12}}{Q_{r1}Q_{r2}} = 1.278 \cdot 10^{-4}$. The total Gibbs free energy of dimerization is:

$$\begin{aligned}
\Delta G &= -RT\left[2 + \ln\left(\frac{\omega_{12}}{\omega_1\omega_2}\right) + \ln\left(\frac{Q_{r12}Q_{t12}}{Q_{r1}Q_{r2}Q_{t1}Q_{t2}}\right)\right] \\
&= -RT\left[2 + \ln\left(\frac{3}{4}\right) + \ln\left(5.325 \cdot 10^{-11}\right)\right] \\
&\approx +22RT
\end{aligned}$$

Where we assumed $\frac{\omega_{12}}{\omega_1\omega_2} = \frac{3}{4}$ as with the triplet state alkoxyl complexes. This 'zero-energy limit' is consistent with typical binding entropies for atmospheric (strongly bound) binding entropies, which are between -29.3 and -39.8 cal mol^{-1}K^{-1}[S9] ($-T\Delta S$ =+(15-20)RT) The detailed balance dissociation rate is then:

$$k_d = k_a c_{gas} \mathrm{e}^{\frac{\Delta G}{RT}} \approx 10^{-10} \frac{\mathrm{cm}^3}{\mathrm{molecule\ s}} \cdot 2.46 \cdot 10^{19} \frac{\mathrm{molecule}}{\mathrm{cm}^3} \mathrm{e}^{22} = 8 \cdot 10^{18}\ \mathrm{s}^{-1} \tag{23}$$

Complex parameter data

The main physical parameters for the model complexes are presented in Table S3. Physical parameters related to angular momentum are presented in Table S4, and model-specific derived parameters are presented in Table S5.

Table S3: Basic physical parameters for the model bimolecular complexes, all in triplet state. The D values are presented with the same number of decimals as in the original source.

Complex	D ($\frac{\text{kcal}}{\text{mol}}$)	μ (amu)	v_c($\frac{\text{m}}{\text{s}}$)	r_e (Å)
$(\text{MetO})_2$	3.32	15.5092	1338.40	3.24081
$(\text{EtO})_2$	5.61	22.51703	1443.90	3.86674
$(\text{ProO})_2$	4.70	29.52486	1154.16	3.82597
$(\text{AceO})_2$	6.85	36.51448	1252.92	3.91762
$(\text{ButO})_2$	4.55	36.53267	1020.88	4.34404
R,R-$(\text{BuOHO})_2$	13.42	44.53013	1588.04	4.11902
R,S-$(\text{BuOHO})_2$	8.02	44.53013	1227.64	4.30456
R-alkoxy,R-nitroxy-α-pin	10.71	107.054	914.96	5.24154
R-alkoxy,S-nitroxy-α-pin	10.18	107.054	892.04	5.33482
S-alkoxy,R-nitroxy-α-pin	18.19	107.054	1192.41	5.46434
S-alkoxy,S-nitroxy-α-pin	10.83	107.054	920.08	5.0185
$(\alpha\text{-pin-O}_3\text{−RO})_2$	13.682	91.55109	1118.29	4.80976
MetO-EtO	4.570	18.36738	1442.93	3.73121
MetO-ProO	3.704	20.33603	1234.56	3.6467
MetO-AceO	3.336	21.77125	1132.35	4.46197
MetO-ProOHO	7.872	21.94699	1732.47	3.63844
MetO-BuOHO	6.993	23.00581	1594.86	3.98679
EtO-ProO	5.221	25.54912	1307.67	3.74744
EtO-AceO	5.983	27.85623	1340.63	3.63135
EtO-ProOHO	9.486	28.14458	1679.40	3.6674
EtO-BuOHO	8.617	29.90988	1552.68	3.98637
ProO-AceO	5.835	32.64978	1222.90	3.95625
ProO-ProOHO	8.980	33.04662	1507.95	3.85528
ProO-BuOHO	8.326	35.50728	1400.78	4.07732
AceO-ProOHO	9.591	37.01154	1472.56	3.99009
AceO-BuOHO	9.898	40.12591	1436.72	4.02101

Table S4: Rotational temperatures and related quantities for the bimolecular complexes. PM (K) is the rotational temperature for two point masses with the same value of μ and r_e as the complex in question. κ is Ray's asymmetry parameter.[S10] $\sqrt{\frac{B+C}{\mu}}$ is the primary rotational constant expressed in units of l, presented to give an idea of the accuracy of the assumption that the probability distribution is continuous.

Complex	$\frac{A}{k}$ (K)	$\frac{B}{k}$ (K)	$\frac{C}{k}$ (K)	PM (K)	κ	$\sqrt{\frac{B+C}{\mu}}$ ($\frac{m}{s}$)
$(MetO)_2$	0.68021	0.14316	0.12206	0.1489	-0.924	11.92
$(EtO)_2$	0.29024	0.06030	0.05575	0.07204	-0.961	6.55
$(ProO)_2$	0.13227	0.04479	0.04219	0.05612	-0.942	4.95
$(AceO)_2$	0.09690	0.03196	0.03033	0.04328	-0.951	3.77
$(ButO)_2$	0.11486	0.02717	0.02677	0.03518	-0.991	3.50
R,R-$(BuOHO)_2$	0.06198	0.02234	0.02226	0.0321	-0.996	2.89
R,S-$(BuOHO)_2$	0.06246	0.02151	0.01955	0.0294	-0.909	2.77
R-alkoxy,R-nitroxy-α-pin	0.01449	0.00519	0.00457	0.00825	-0.875	0.87
R-alkoxy,S-nitroxy-α-pin	0.00976	0.00565	0.00485	0.00796	-0.671	0.90
S-alkoxy,R-nitroxy-α-pin	0.01269	0.00511	0.00452	0.00759	-0.856	0.86
S-alkoxy,S-nitroxy-α-pin	0.01020	0.00645	0.00478	0.009	-0.384	0.93
(α-pin-O_3−RO$)_2$	0.01196	0.00801	0.00585	0.01145	-0.292	1.12
MetO-EtO	0.47874	0.08464	0.07399	0.09485	-0.947	8.47
MetO-ProO	0.25874	0.07157	0.06609	0.08969	-0.943	7.50
MetO-AceO	0.30333	0.04373	0.03959	0.05596	-0.969	5.64
MetO-ProOHO	0.19868	0.06124	0.05053	0.08348	-0.856	6.51
MetO-BuOHO	0.15768	0.04280	0.03578	0.06633	-0.885	5.33
EtO-ProO	0.17826	0.05585	0.04985	0.0676	-0.906	5.87
EtO-AceO	0.14638	0.04909	0.04483	0.06603	-0.916	5.29
EtO-ProOHO	0.14539	0.04732	0.04191	0.06407	-0.895	5.13
EtO-BuOHO	0.11445	0.03510	0.03036	0.05103	-0.887	4.27
ProO-AceO	0.11787	0.03574	0.03462	0.04746	-0.973	4.23
ProO-ProOHO	0.11618	0.03743	0.03518	0.04938	-0.944	4.27
ProO-BuOHO	0.09607	0.02927	0.02860	0.04109	-0.980	3.68
AceO-ProOHO	0.09847	0.03095	0.02907	0.04116	-0.946	3.67
AceO-BuOHO	0.06804	0.02897	0.02385	0.03738	-0.768	3.31

As touched on in the main article, the variance of the minimal escape velocity on l was determined numerically and fit to the following empirical second-order polynomial, where the parameters α and β are presented in Table S5:

$$v_c(l) = v_c(0) - \alpha l - \beta l^2 \tag{24}$$

Table S5: Parameters derived from equation 24. The vertical lines separate level-of-theory-specific parameters from general ones.

Complex	v_c ($\frac{m}{s}$)	$\sqrt{\langle l^2 \rangle}$ ($\frac{m}{s}$)	$v_c(\sqrt{\langle l^2 \rangle})$	α	β ($\frac{s}{m}$)	Fit R^2
$(MetO)_2$	1338.40	599.1	1215.47	0.00299	0.000330	0.9999
$(EtO)_2$	1443.90	522.9	1356.21	0.00871	0.000301	1.0000
$(ProO)_2$	1154.16	465.5	1066.88	0.00182	0.000384	0.9999
$(AceO)_2$	1252.92	434.4	1182.73	0.00189	0.00354	0.9999
$(ButO)_2$	1020.88	420.8	941.13	0.00000	0.000437	0.9996
R,R-$(BuOHO)_2$	1588.04	400.4	1539.61	0.00675	0.000275	0.9999
R,S-$(BuOHO)_2$	1227.64	399.3	1166.88	0.00000	0.000363	0.9998
R-alkoxy,R-nitroxy-α-pin	914.96	279.8	873.53	0.00000	0.000486	0.9998
R-alkoxy,S-nitroxy-α-pin	892.04	265.0	855.09	0.00000	0.000499	0.9999
S-alkoxy,R-nitroxy-α-pin	1192.41	270.1	1162.82	0.00191	0.000371	0.9999
S-alkoxy,S-nitroxy-α-pin	920.08	272.4	882.15	0.00000	0.000484	0.9998
(α-pin-O_3−RO$)_2$	1118.29	299.1	1080.37	0.00032	0.000398	0.9998
MetO-EtO	1442.93	568.2	1339.59	0.00469	0.000305	0.9999
MetO-ProO	1234.56	563.6	1116.94	0.00308	0.000358	0.9999
MetO-AceO	1132.35	553.1	1009.14	0.00000	0.000394	0.9994
MetO-ProOHO	1732.47	580.9	1641.86	0.00754	0.000252	0.9999
MetO-BuOHO	1594.86	603.2	1489.24	0.00198	0.000278	0.9999
EtO-ProO	1307.67	498.2	1219.8	0.00478	0.000336	0.9999
EtO-AceO	1340.63	500.3	1254.13	0.00638	0.000326	0.9999
EtO-ProOHO	1679.40	503.0	1608.62	0.00791	0.000259	0.9999
EtO-BuOHO	1552.68	508.3	1474.56	0.00551	0.000282	0.9999
ProO-AceO	1222.90	452.6	1145.3	0.00305	0.000361	0.9999
ProO-ProOHO	1507.95	451.7	1443.99	0.00764	0.000289	0.9999
ProO-BuOHO	1400.78	445.3	1333.88	0.00596	0.000313	0.9999
AceO-ProOHO	1472.56	428.7	1413.02	0.00602	0.000297	0.9999
AceO-BuOHO	1436.72	418.2	1378.44	0.00683	0.000304	0.9999
$(MetO)_2$ (Trial $V(r)$)	1339.40			0.00000	0.000402	0.9941
$(AceO)_2$ (Trial $V(r)$)	1252.92			0.00000	0.000430	0.9928
MetO-ProOHO (Trial $V(r)$)	1732.47			0.03000	0.000221	0.9970

Table S6: Parameters related to centrifugal distortion.

Complex	Θ ($\cdot 10^{-4} \frac{Ks^2}{m^2}$)	Θ_D ($\cdot 10^{-12} \frac{Ks^4}{m^4}$)	l_c ($\frac{m}{s}$)	N_D
$(MetO)_2$	8.307	11.472	1871.07	0.98992
$(EtO)_2$	10.906	11.703	2018.56	0.99406
$(ProO)_2$	13.759	22.232	1613.51	0.9929
$(AceO)_2$	15.801	20.119	1751.57	0.99515
$(ButO)_2$	16.840	34.406	1427.19	0.99266
R,R-$(BuOHO)_2$	18.600	14.230	2220.06	0.99754
R,S-$(BuOHO)_2$	18.703	24.075	1716.23	0.99586
R-alkoxy,R-nitroxy-α-pin	38.085	74.755	1279.11	0.99691
R-alkoxy,S-nitroxy-α-pin	42.458	97.747	1247.06	0.99675
S-alkoxy,R-nitroxy-α-pin	40.86	50.663	1666.98	0.99818
S-alkoxy,S-nitroxy-α-pin	40.19	82.324	1286.26	0.99694
(α-pin-O_3−RO$)_2$	33.33	44.819	1563.36	0.99758
MetO-EtO	9.236	10.304	2017.20	0.99269
MetO-ProO	9.385	13.127	1725.91	0.99100
MetO-AceO	9.747	15.720	1583.02	0.99007
MetO-ProOHO	8.835	5.474	2421.98	0.99578
MetO-BuOHO	8.195	5.301	2229.61	0.99525
EtO-ProO	12.012	15.255	1828.12	0.99361
EtO-AceO	11.913	13.093	1874.19	0.99444
EtO-ProOHO	11.785	8.082	2347.79	0.99651
EtO-BuOHO	11.538	8.527	2170.63	0.99615
ProO-AceO	14.553	20.035	1709.60	0.99429
ProO-ProOHO	14.612	13.124	2108.09	0.99631
ProO-BuOHO	15.037	14.991	1958.28	0.99601
AceO-ProOHO	16.226	15.153	2058.63	0.99654
AceO-BuOHO	17.046	16.205	2008.52	0.99665

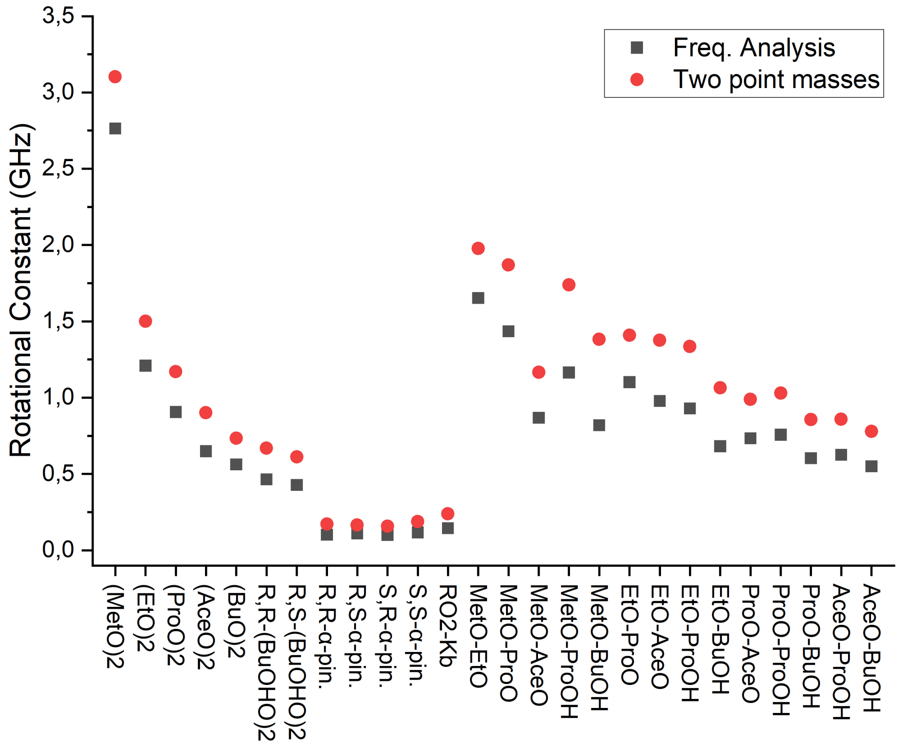

Figure S5: Rotational constants determined for equilibrium geometry using frequency analysis compared with the same values for two point masses. Note that the point mass result is always a slight overestimate.

v,l Probability distributions visualised

This section includes visualized probability distributions for all bimolecular complexes. All non-dissociative trajectories, that is, v, l-values for which $v < v_c(l)$, are cut out from the figures, just like in the code.

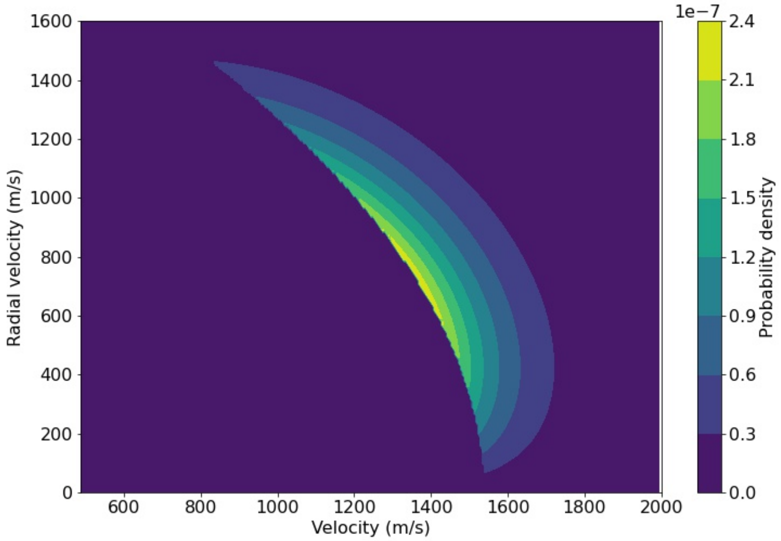

Figure S6: v, l-probability distribution for $(MetO)_2$.

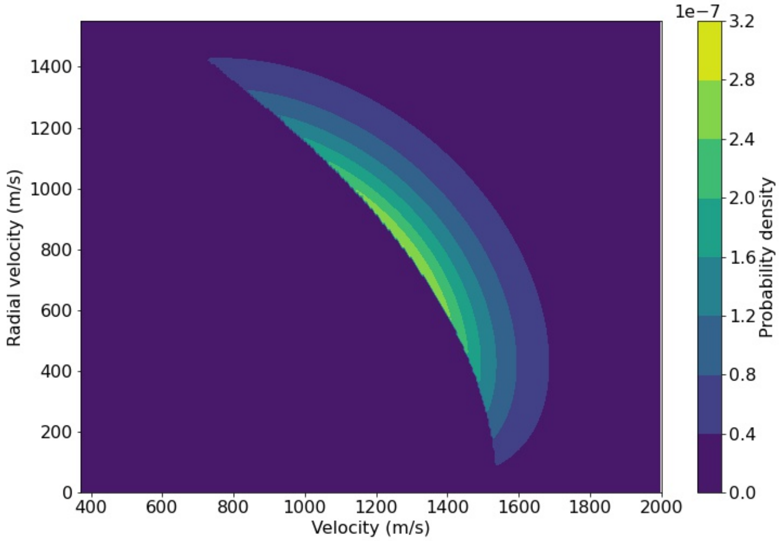

Figure S7: v, l-probability distribution for $(MetO)_2$ with Equation 10a.

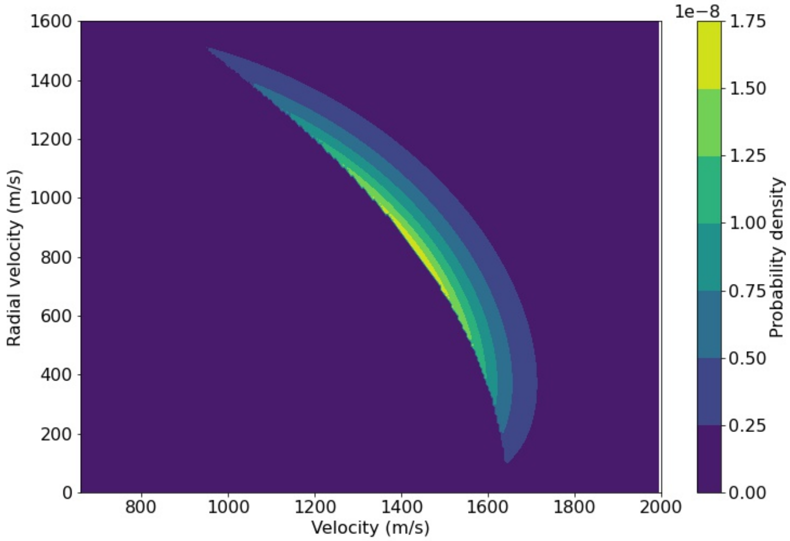

Figure S8: v, l-probability distribution for $(\mathrm{EtO})_2$.

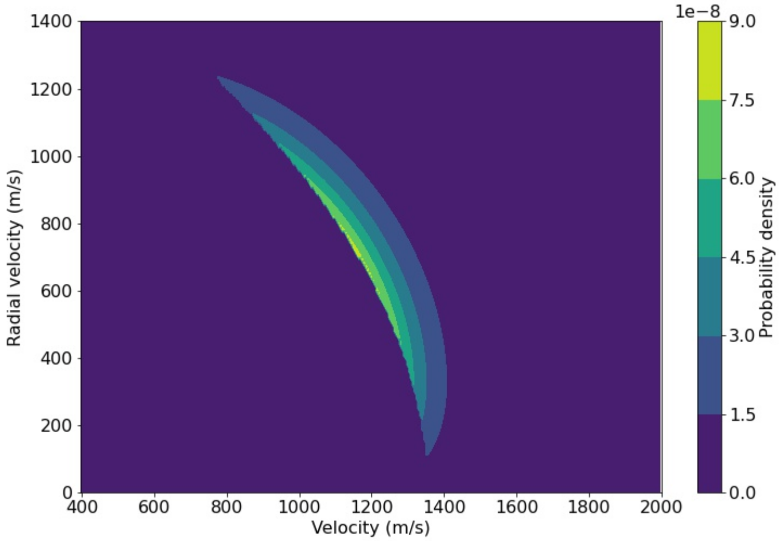

Figure S9: v, l-probability distribution for $(\mathrm{ProO})_2$.

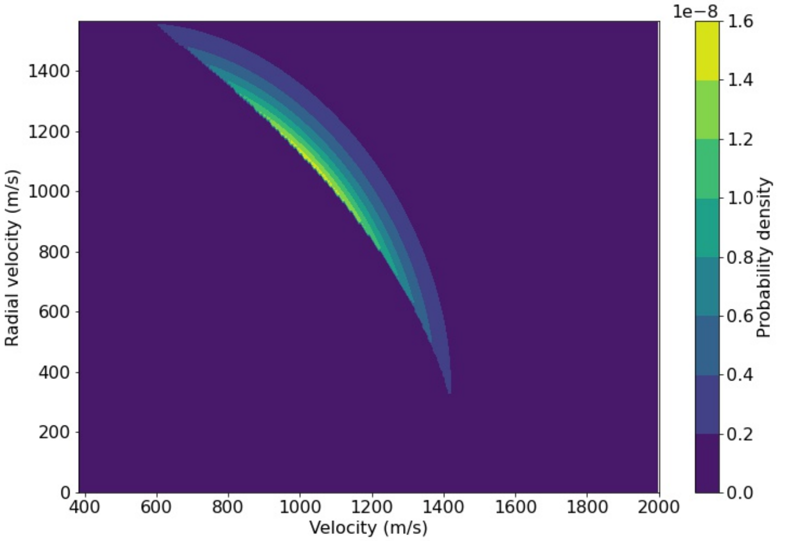

Figure S10: v, l-probability distribution for $(\mathrm{AceO})_2$.

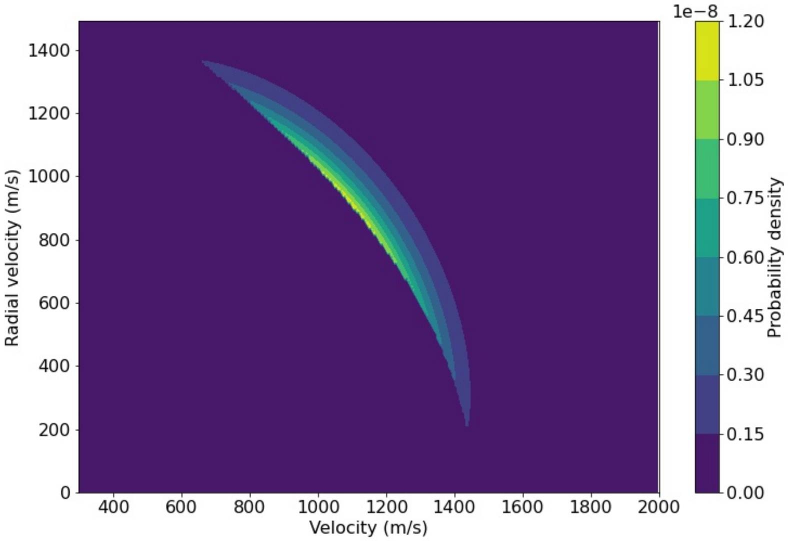

Figure S11: v, l-probability distribution for $(\mathrm{AceO})_2$ with Equation 10b.

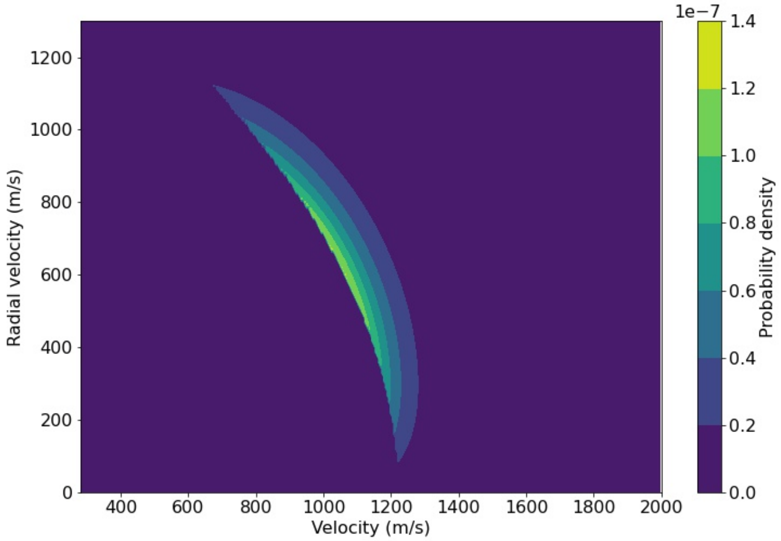

Figure S12: v, l-probability distribution for $(\mathrm{ButO})_2$.

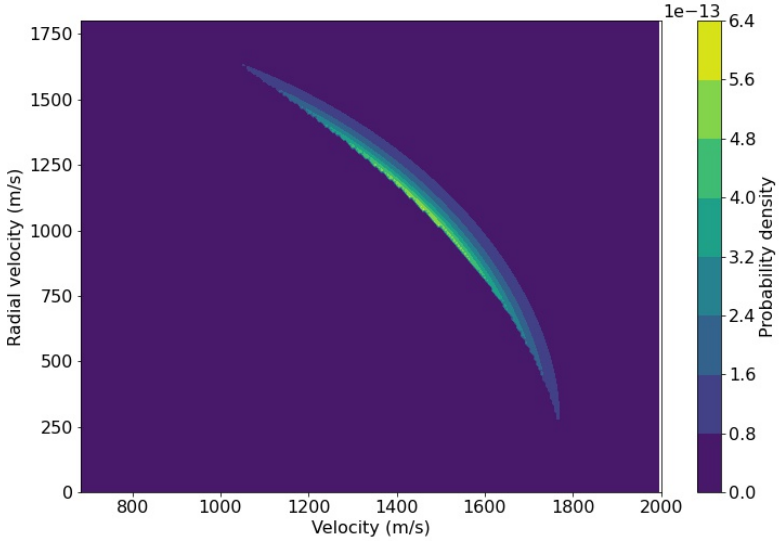

Figure S13: v, l-probability distribution for R,R-$(\mathrm{BuOHO})_2$.

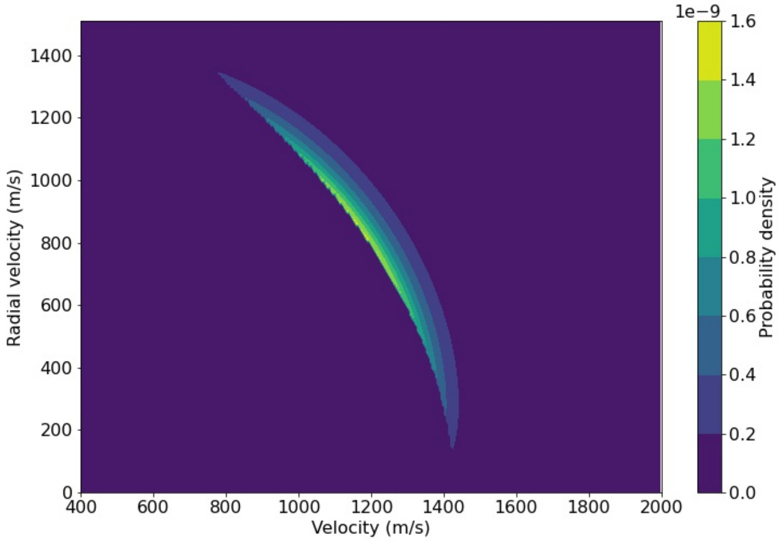

Figure S14: v, l-probability distribution for R,S-$(BuOHO)_2$.

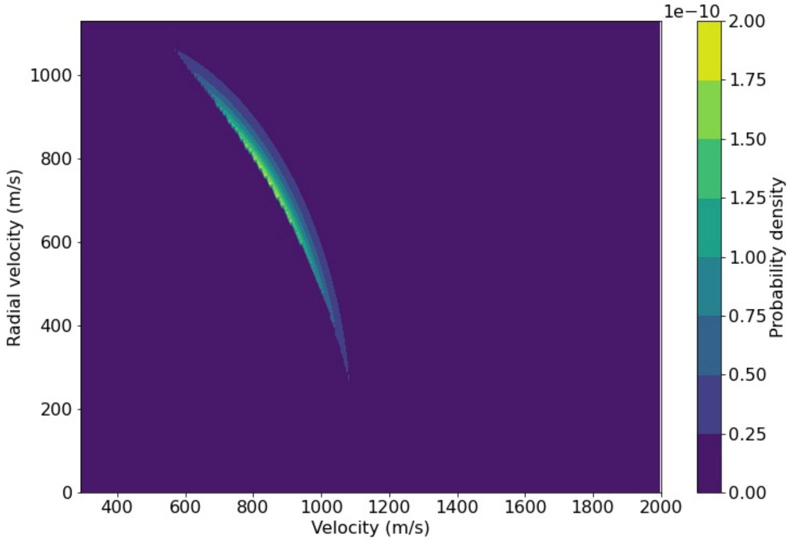

Figure S15: v, l-probability distribution for R-alkoxy,R-nitroxy-α-pin.

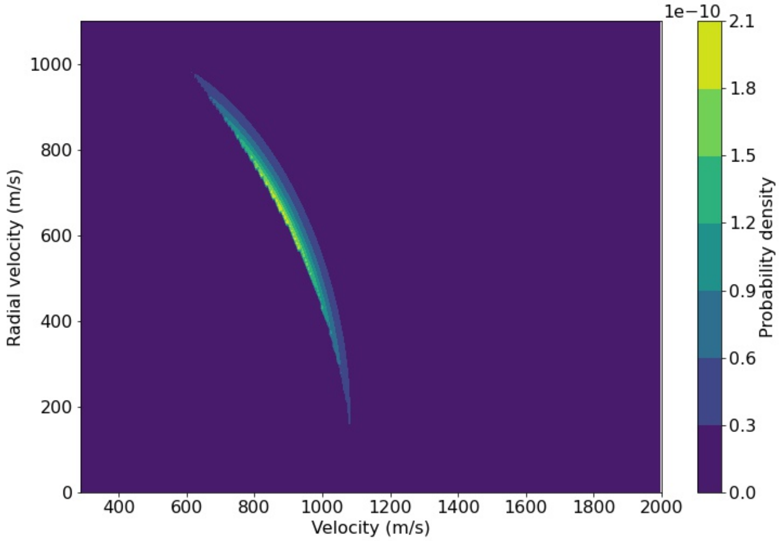

Figure S16: v, l-probability distribution for R-alkoxy,S-nitroxy-α-pin.

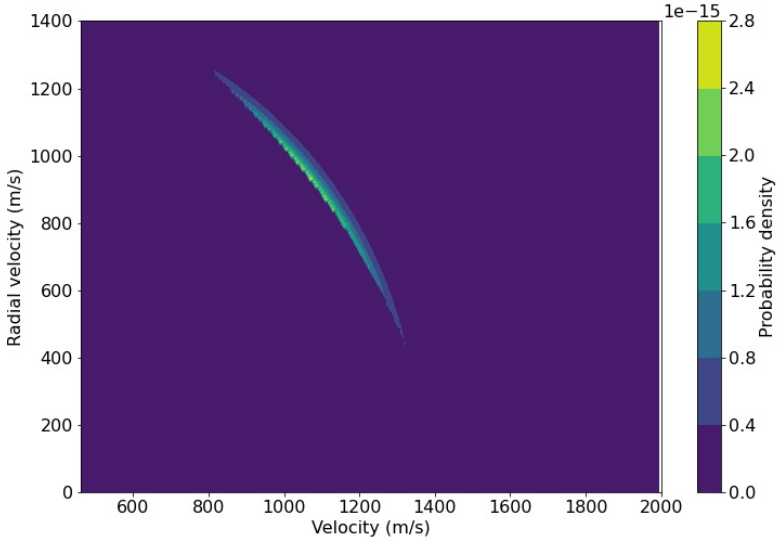

Figure S17: v, l-probability distribution for S-alkoxy,R-nitroxy-α-pin.

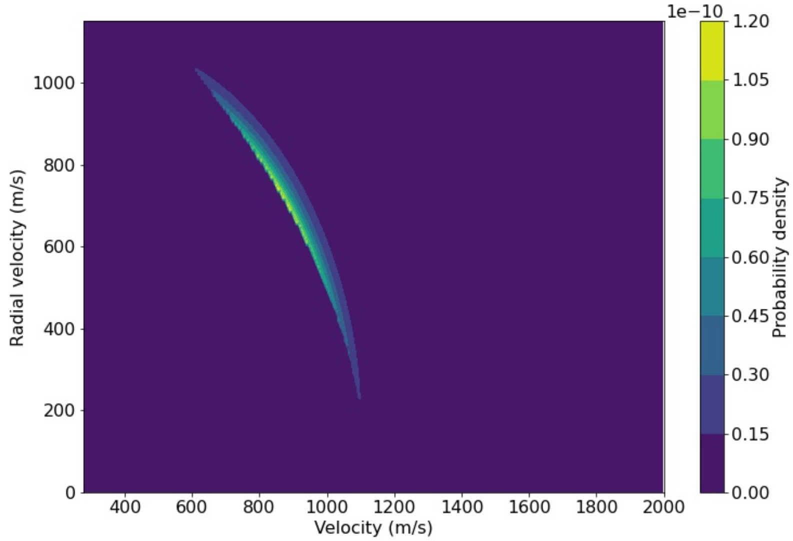

Figure S18: v, l-probability distribution for S-alkoxy,S-nitroxy-α-pin.

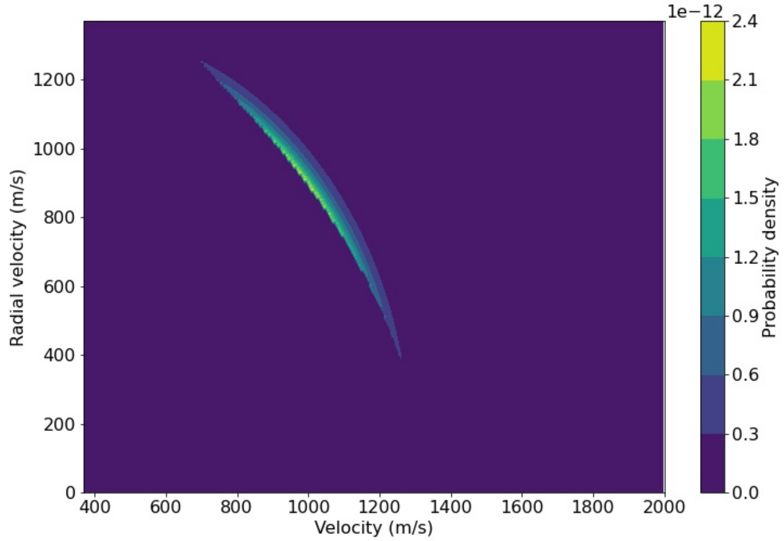

Figure S19: v, l-probability distribution for (α-pin-$O_3$$-RO)_2$.

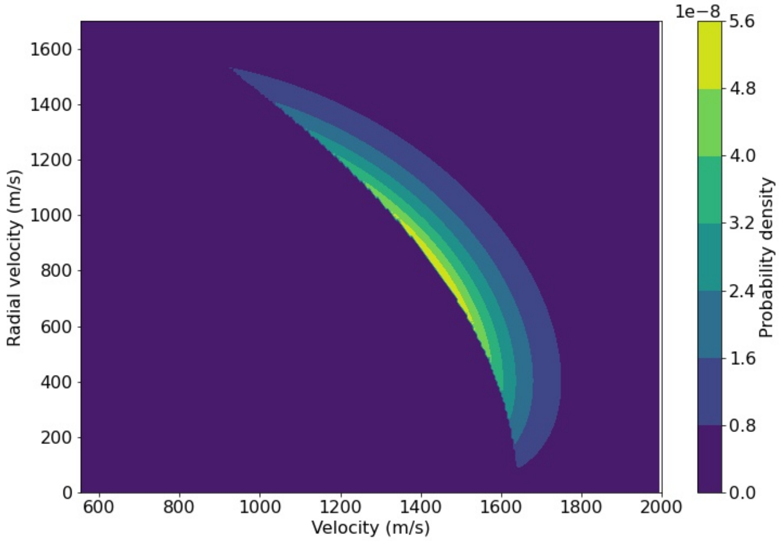

Figure S20: v, l-probability distribution for MetO-EtO.

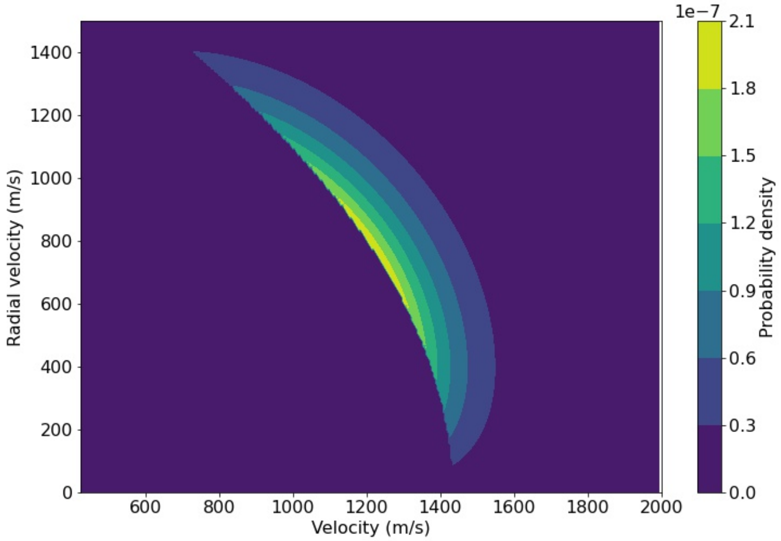

Figure S21: v, l-probability distribution for MetO-ProO.

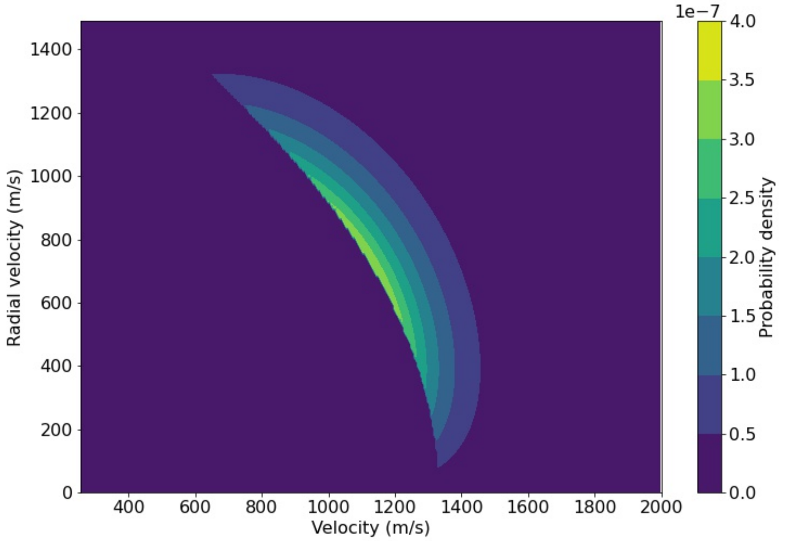

Figure S22: v, l-probability distribution for MetO-AceO.

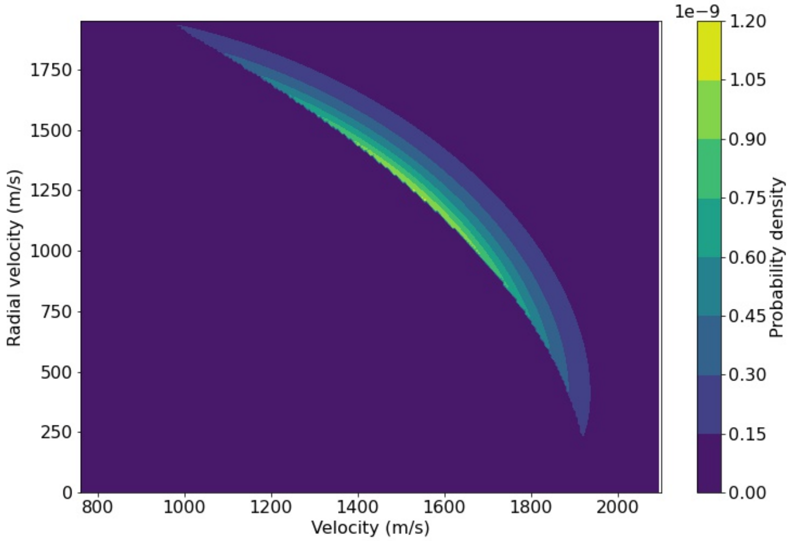

Figure S23: v, l-probability distribution for MetO-ProOHO.

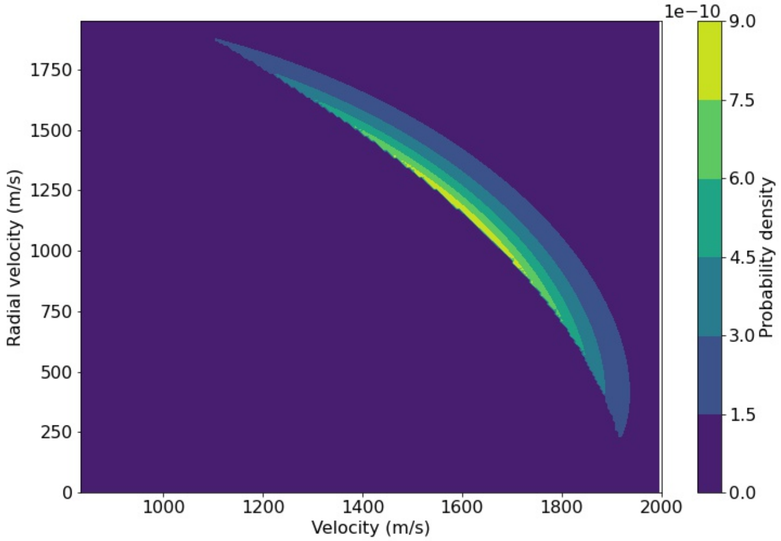

Figure S24: v, l-probability distribution for MetO-ProOHO with Equation 10c.

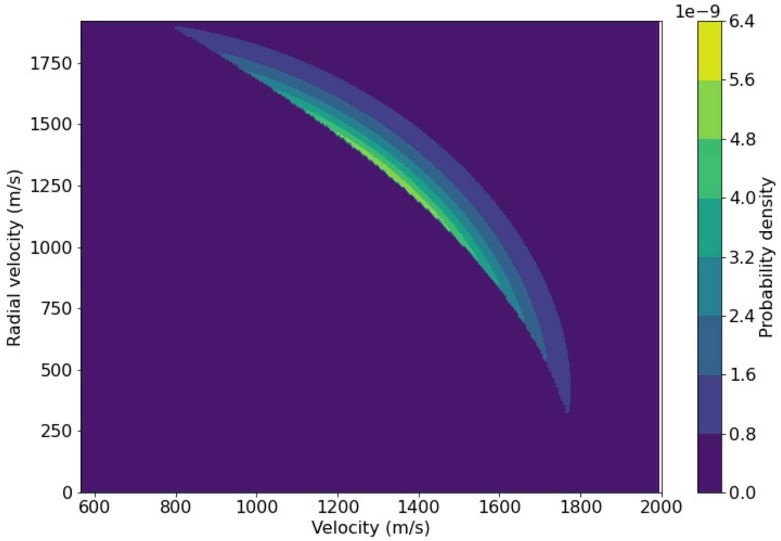

Figure S25: v, l-probability distribution for MetO-BuOHO.

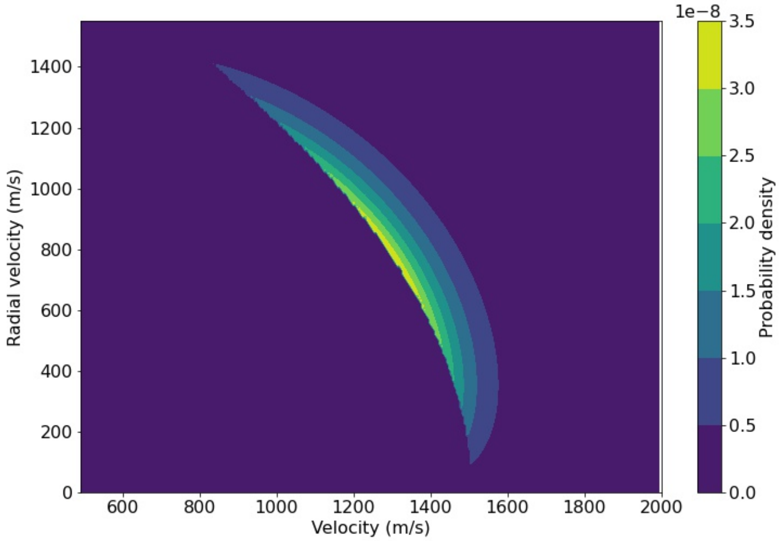

Figure S26: v, l-probability distribution for EtO-ProO.

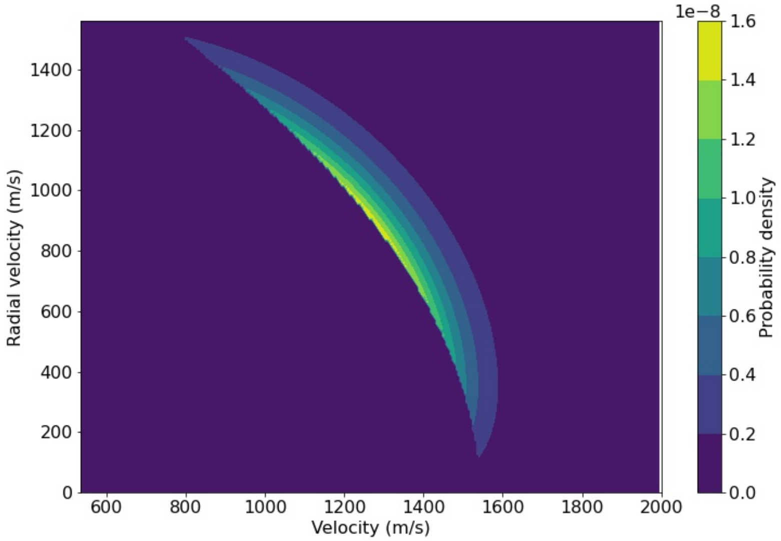

Figure S27: v, l-probability distribution for EtO-AceO.

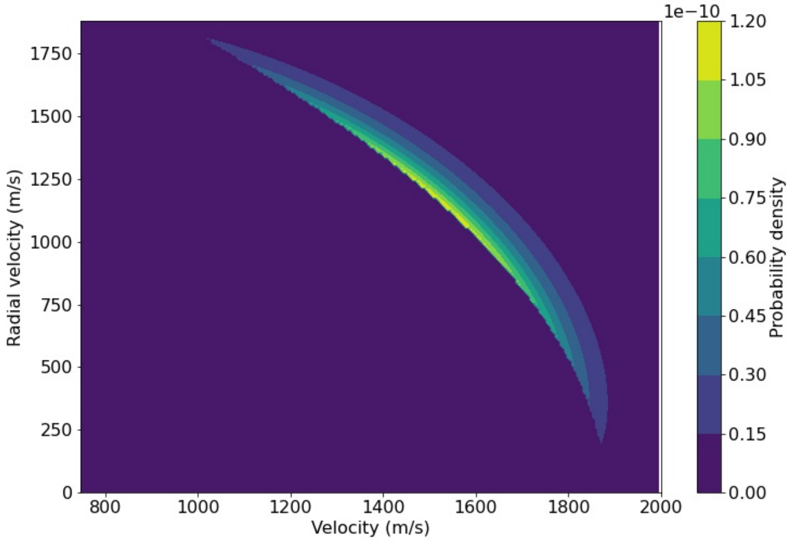

Figure S28: v, l-probability distribution for EtO-ProOHO.

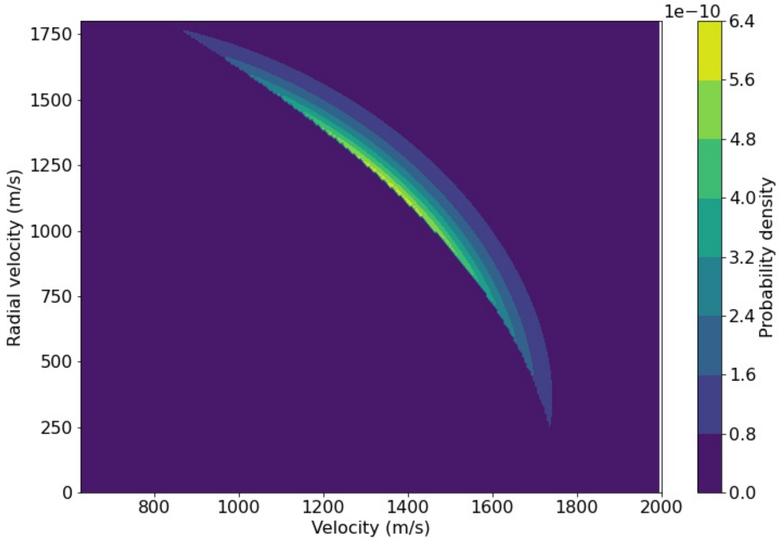

Figure S29: v, l-probability distribution for EtO-BuOHO.

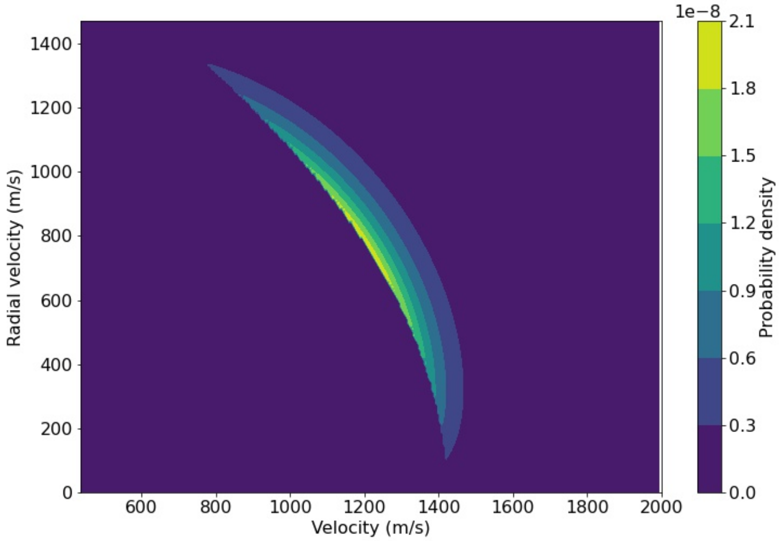

Figure S30: v, l-probability distribution for ProO-AceO.

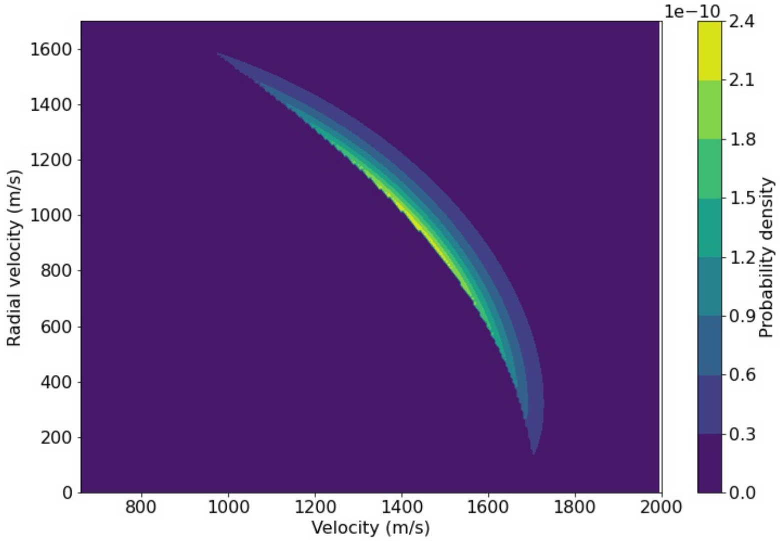

Figure S31: v, l-probability distribution for ProO-ProOHO.

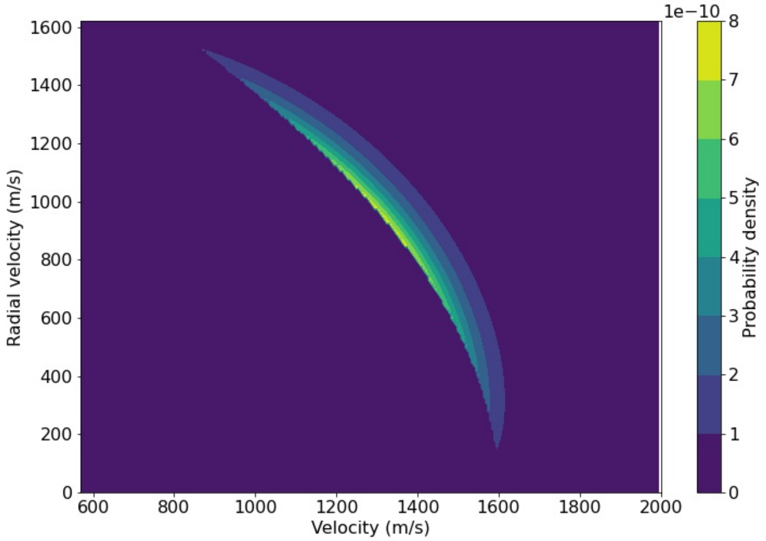

Figure S32: v, l-probability distribution for ProO-BuOHO.

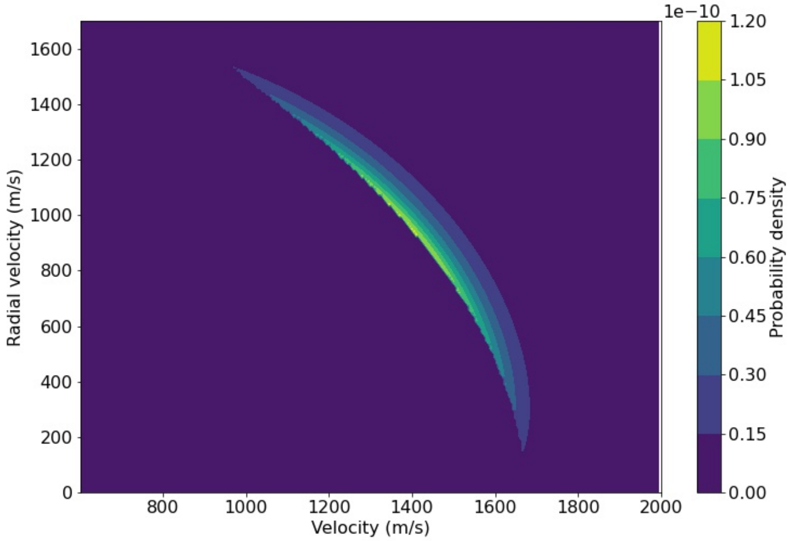

Figure S33: v, l-probability distribution for AceO-ProOHO.

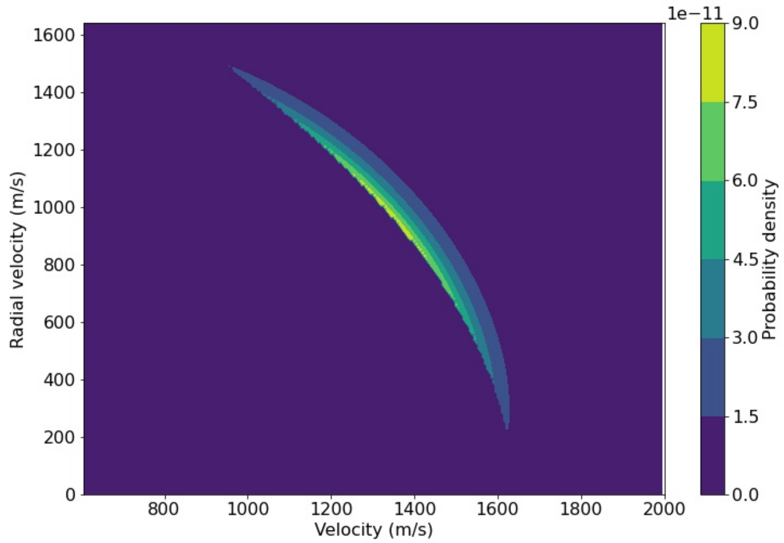

Figure S34: v, l-probability distribution for AceO-BuOHO.

xyz geometries used for parameter determination

The alkoxy radical complex geometries are from sources.[S11–S14] Citations are presented in the table captions. r_e-parameters were determined using the formula for centre of mass:

$$C\vec{O}M_i = \frac{1}{m_i}\sum_j m_j \vec{r}_j \tag{25a}$$

$$r_d = \left| C\vec{O}M_2 - C\vec{O}M_1 \right| \tag{25b}$$

In the equation i represents a molecule and j represents an atom. The geometries are presented below. Horizontal lines are used to show where one molecule ends and the other begins.

Table S7: $(MetO)_2$[S11]

Atom	x	y	z
C	1.716466	0.516739	0.029196
H	0.707752	0.980722	-0.001817
H	2.234322	0.836632	-0.883685
H	2.207142	0.902649	0.931621
O	1.486758	-0.817757	0.075767
C	-1.674008	-0.52482	-0.031268
H	-0.903425	-0.987526	-0.663719
H	-2.675298	-0.906714	-0.256568
H	-1.408631	-0.837926	0.995538
O	-1.591919	0.830522	-0.036276

Table S8: $(EtO)_2$[S11]

Atom	x	y	z
H	-3.709067	0.286887	-0.604243
C	-3.068822	0.404742	0.269153
H	-3.235278	1.396721	0.689885
H	-3.359397	-0.338124	1.011096
C	-1.610836	0.229358	-0.113244
H	-1.27389	1.005302	-0.826308
H	-0.93091	0.405654	0.740867
O	-1.281545	-0.969673	-0.656842
H	3.210804	-0.335352	1.231933
C	2.361929	-0.817621	0.748146
H	2.6768	-1.80196	0.400991
H	1.570953	-0.946997	1.486139
C	1.866585	0.02201	-0.419034
H	2.608782	0.098185	-1.229144
H	0.995619	-0.480646	-0.893282
O	1.388632	1.247605	-0.091823

Table S9: $(ProO)_2$[S11]

Atom	x	y	z
H	-2.25444	-1.60805	-1.18600
C	-1.99069	-1.40574	-0.14845
H	-2.83655	-1.68103	0.48267
H	-1.13661	-2.02400	0.12211
C	-1.65723	0.07484	0.04035
H	-0.76831	0.28399	-0.59739
C	-2.78194	1.01616	-0.38641
H	-3.67038	0.83488	0.21998
H	-3.04308	0.85407	-1.43200
H	-2.47839	2.05317	-0.25497
O	-1.16513	0.35248	1.27626
H	2.86193	1.56544	0.96475
C	2.09244	1.39080	0.21185
H	1.11518	1.50926	0.68018
H	2.20265	2.14180	-0.56947
C	2.24059	-0.00795	-0.37406
H	3.25948	-0.16678	-0.75680
C	1.98956	-1.09973	0.69839
H	0.99743	-0.97532	1.12937
H	2.73752	-0.97312	1.48005
H	2.08597	-2.09325	0.26683
O	1.33165	-0.27655	-1.35611

Table S10: $(AceO)_2$[S11]

Atom	x	y	z
H	-1.856455	2.335695	-0.864479
C	-2.2595	1.508161	-0.288495
H	-2.141391	1.689142	0.780794
H	-3.329175	1.399332	-0.469084
C	-1.557919	0.239403	-0.653263
C	-1.935437	-1.024033	0.141574
H	-1.677606	-1.922239	-0.436781
H	-1.219225	-0.976137	0.993056
O	-0.703547	0.158412	-1.499719
O	-3.165001	-1.027876	0.672755
H	1.856538	2.335732	0.864112
C	2.259558	1.508097	0.288254
H	2.14141	1.688897	-0.781062
H	3.329239	1.399291	0.468822
C	1.557986	0.239405	0.653266
C	1.935448	-1.024161	-0.141392
H	1.677589	-1.922272	0.437099
H	1.219234	-0.976365	-0.992878
O	0.703617	0.158573	1.499741
O	3.165008	-1.028129	-0.67258

Table S11: $(ButO)_2$[S11]

Atom	x	y	z
H	0.73019	1.353986	-0.871917
C	1.793303	1.347794	-0.635426
H	2.358667	1.490314	-1.557001
H	2.01247	2.181996	0.031581
C	2.183748	0.026853	0.033713
C	3.684236	-0.040737	0.333266
H	4.264498	0.066721	-0.583657
H	3.934041	-0.992169	0.8009
H	3.96652	0.76373	1.012532
C	1.770842	-1.164804	-0.874654
H	2.335929	-1.07988	-1.802845
H	0.707082	-1.12347	-1.099526
H	2.011438	-2.111025	-0.394001
O	1.448864	-0.188326	1.169437
H	-3.785047	0.987183	-1.023936
C	-3.595545	0.052519	-0.500317
H	-4.248655	-0.011501	0.369631
H	-3.800943	-0.783708	-1.165458
C	-2.128219	-0.000866	0.007993
C	-1.869797	-1.329629	0.722693
H	-2.510641	-1.424429	1.599993
H	-0.832068	-1.373902	1.051249
H	-2.065789	-2.164387	0.050484
C	-1.846749	1.19631	0.91995
H	-2.486536	1.162705	1.802521
H	-2.029145	2.128466	0.386266
H	-0.807542	1.169325	1.245202
O	-1.40764	0.085579	-1.155143

Table S12: R,R-$(BuOHO)_2$[S11]

Atom	x	y	z
H	-4.447040	1.126650	0.095830
C	-3.482050	1.628690	-0.002020
H	-3.246440	1.712420	-1.062650
H	-3.597180	2.638740	0.388780
C	-2.390980	0.885330	0.754440
H	-2.566150	0.959620	1.829280
H	-1.414990	1.327120	0.555030
C	-2.321190	-0.606740	0.424790
H	-3.306950	-1.096020	0.505500
C	-1.981760	-0.811490	-1.173650
H	-2.913010	-0.513360	-1.655600
H	-1.805650	-1.878600	-1.307240
O	-0.965440	-0.018730	-1.616390
H	-0.104900	-0.449880	-1.436020
O	-1.372040	-1.281480	1.052130
H	4.447060	1.126600	-0.095910
C	3.482070	1.628660	0.001840
H	3.246430	1.712540	1.062460
H	3.597230	2.638660	-0.389090
C	2.391010	0.885220	-0.754560
H	2.566220	0.959370	-1.829400
H	1.415020	1.327050	-0.555240
C	2.321190	-0.606810	-0.424710
H	3.306940	-1.096120	-0.505340
C	1.981740	-0.811330	1.173760
H	2.912980	-0.513090	1.655680
H	1.805660	-1.878430	1.307500
O	0.965410	-0.018530	1.616370
H	0.104890	-0.449710	1.436010
O	1.372030	-1.281610	-1.051970

Table S13: R,S-$(BuOHO)_2$[S11]

Atom	x	y	z
H	-3.733163	1.328842	0.773103
C	-3.646984	0.444658	1.403512
H	-2.946003	0.684191	2.204149
H	-4.619688	0.255971	1.855836
C	-3.183764	-0.774097	0.617935
H	-3.869558	-1.00164	-0.200542
H	-3.168999	-1.650437	1.268605
C	-1.776121	-0.626239	0.039366
C	-1.748016	0.629304	-1.055833
H	-2.774712	0.695162	-1.412021
H	-1.087276	0.331656	-1.868559
O	-1.374113	1.797013	-0.454732
H	-0.400676	1.879596	-0.515377
H	-1.061114	-0.284223	0.804577
O	-1.350458	-1.665524	-0.659217
H	3.733078	-1.328815	0.773117
C	3.646996	-0.4446	1.403499
H	2.946079	-0.684068	2.204212
H	4.619751	-0.25594	1.855724
C	3.183743	0.774137	0.617917
H	3.869534	1.001698	-0.200558
H	3.168936	1.650481	1.26858
C	1.776101	0.626165	0.039359
C	1.748099	-0.629347	-1.055879
H	2.774877	-0.695322	-1.411816
H	1.087622	-0.331585	-1.86878
O	1.373902	-1.797032	-0.454914
H	0.400446	-1.879515	-0.51563
H	1.061151	0.284057	0.804581
O	1.350319	1.665436	-0.659175

Table S14: R-alkoxy,R-nitroxy-α-pin[S12]

Atom	x	y	z	Atom	x	y	z
C	-2.104444	-0.106314	0.924843	C	2.138858	0.179468	0.875478
C	-2.99172	0.541302	-1.508888	C	3.029854	-0.610337	-1.498651
H	-2.541222	0.22391	-2.454246	H	2.618569	-0.31165	-2.467642
H	-3.408264	1.540179	-1.672457	H	3.401788	-1.633572	-1.611384
C	-4.094298	-0.441531	-1.115736	C	4.168731	0.334633	-1.114302
H	-4.823588	-0.557716	-1.924329	H	4.913116	0.396649	-1.915085
C	-3.440285	-1.707996	-0.518585	C	3.564716	1.646049	-0.559573
H	-2.479864	-2.02098	-0.933804	H	2.622598	1.992257	-0.995489
H	-4.105242	-2.571187	-0.498651	H	4.267321	2.479066	-0.555471
C	-3.424674	-0.9439	0.83093	C	3.494232	0.912998	0.8071
H	-3.555295	-1.506375	1.759264	H	3.632522	1.498286	1.721288
C	-4.646619	-0.111342	0.301354	C	4.686147	0.022111	0.320917
C	-5.947571	-0.863962	0.609944	C	6.021292	0.715244	0.618889
H	-5.897412	-1.929879	0.376667	H	6.02723	1.774301	0.350515
H	-6.772335	-0.434869	0.030304	H	6.830634	0.227172	0.064631
H	-6.197624	-0.766838	1.672042	H	6.254902	0.642496	1.686859
C	-4.862987	1.3558	0.664768	C	4.820854	-1.442471	0.735798
H	-4.008599	2.00097	0.456505	H	3.937482	-2.050097	0.532401
H	-5.115549	1.461737	1.72511	H	5.046391	-1.523179	1.80479
H	-5.708769	1.743275	0.084961	H	5.655865	-1.89627	0.189497
C	-1.871259	0.637041	-0.44342	C	1.879117	-0.606664	-0.462132
C	-2.059661	0.777495	2.180421	C	1.981932	-0.666055	2.169621
H	-1.061233	1.206389	2.286054	H	0.960607	-1.040426	2.2561
H	-2.269567	0.145944	3.047028	H	2.218721	-0.032468	3.026231
H	-2.777349	1.594938	2.1468	H	2.661185	-1.516523	2.144003
O	-1.155337	-1.089345	1.070548	O	1.150086	1.122633	1.047332
H	-0.96049	0.206615	-0.846561	H	1.007283	-0.132476	-0.9031
O	-1.598104	2.026906	-0.143731	O	1.509465	-1.963368	-0.122194
N	-0.569592	2.613608	-0.852574	N	0.494087	-2.522337	-0.872237
O	-0.019313	1.971405	-1.721675	O	0.038849	-1.887159	-1.799026
O	-0.350348	3.744569	-0.509456	O	0.187383	-3.622888	-0.499522

Table S15: R-alkoxy,S-nitroxy-α-pin[S12]

Atom	x	y	z	Atom	x	y	z
C	-2.037003	-0.100271	0.535997	C	2.860119	-0.685722	-0.618953
C	-4.053382	0.315767	-1.134275	C	2.380925	1.12625	1.259548
H	-4.210885	1.261131	-1.661342	H	2.823725	1.022592	2.254565
H	-5.042917	-0.118997	-0.966256	H	1.427728	1.647482	1.384177
C	-3.204377	-0.609578	-2.006572	C	3.331048	1.935202	0.377342
H	-3.667122	-0.747819	-2.989345	H	3.515494	2.923173	0.812118
C	-1.731318	-0.149114	-1.936639	C	4.538873	1.048566	0.004228
H	-1.546401	0.92495	-1.839092	H	4.871403	0.317568	0.747523
H	-1.119432	-0.535295	-2.752383	H	5.405599	1.612537	-0.340771
C	-1.636076	-1.0057	-0.646478	C	3.658865	0.518506	-1.155852
H	-0.69775	-1.51199	-0.407377	H	4.143262	0.237988	-2.096572
C	-2.794066	-1.887687	-1.219201	C	2.886046	1.881059	-1.113346
C	-2.215345	-2.979119	-2.128135	C	3.605266	2.926413	-1.974077
H	-1.45353	-2.606094	-2.816854	H	4.687681	2.935429	-1.825151
H	-3.011663	-3.433975	-2.727423	H	3.228755	3.92879	-1.742261
H	-1.755033	-3.767493	-1.523094	H	3.418583	2.735297	-3.036771
C	-3.837243	-2.542153	-0.315215	C	1.390946	1.976505	-1.418013
H	-4.359454	-1.843887	0.343105	H	0.765996	1.314604	-0.814679
H	-3.379456	-3.315488	0.310354	H	1.19302	1.761836	-2.474459
H	-4.599933	-3.027294	-0.934725	H	1.04417	2.996889	-1.219466
C	-3.399985	0.649746	0.232753	C	2.097006	-0.295997	0.71458
O	-3.1561	2.070972	0.146459	O	2.516394	-1.166832	1.792552
H	-4.103501	0.480335	1.050294	H	1.023889	-0.423756	0.581554
N	-2.913135	2.720241	1.347463	N	1.980946	-2.434641	1.801051
O	-3.144767	2.12646	2.375986	O	1.079285	-2.689551	1.027225
O	-2.524816	3.847101	1.198991	O	2.466734	-3.149784	2.634968
C	-2.076503	-0.889488	1.8785	C	1.891868	-1.245527	-1.703471
H	-2.183122	-0.197564	2.714737	H	1.434774	-2.174189	-1.360325
H	-1.157168	-1.468237	1.983355	H	2.448284	-1.42814	-2.624526
H	-2.926812	-1.570899	1.872094	H	1.109777	-0.510728	-1.893252
O	-1.027545	0.792102	0.769694	O	3.698733	-1.748455	-0.426333

Table S16: S-alkoxy,R-nitroxy-α-pin[S12]

Atom	x	y	z	Atom	x	y	z
C	-2.308286	0.411229	-0.730448	C	2.65045	0.123106	0.780021
C	-3.321675	-0.628545	1.47024	C	4.121292	0.527431	-1.387849
H	-3.152438	-0.439885	2.533736	H	5.13912	0.925443	-1.448873
H	-3.489102	-1.704708	1.358513	H	3.595981	0.859205	-2.28781
C	-4.543337	0.154566	0.987272	C	4.171663	-0.998201	-1.334163
H	-5.386829	0.035637	1.675189	H	4.772208	-1.395446	-2.159141
C	-4.099622	1.589357	0.605721	C	4.525122	-1.423654	0.108818
H	-3.300218	2.047396	1.197066	H	5.201803	-0.769146	0.669451
H	-4.925736	2.29839	0.550117	H	4.899436	-2.444338	0.188073
C	-3.739245	0.995847	-0.779723	C	3.006272	-1.33238	0.397273
H	-3.847724	1.618368	-1.673354	H	2.55751	-2.019263	1.118745
C	-4.816104	-0.104715	-0.52601	C	2.753743	-1.611955	-1.115685
C	-6.210537	0.37296	-0.945987	C	2.725565	-3.118814	-1.393342
H	-6.458594	1.367301	-0.565431	H	3.559412	-3.657658	-0.935125
H	-6.972814	-0.322244	-0.577166	H	2.764257	-3.302166	-2.472734
H	-6.286637	0.405689	-2.038259	H	1.796061	-3.548586	-1.007346
C	-4.603509	-1.509942	-1.081958	C	1.551182	-0.976367	-1.805027
H	-3.609515	-1.918768	-0.883347	H	1.498333	0.10753	-1.684007
H	-4.740536	-1.512381	-2.167883	H	0.619302	-1.396951	-1.414597
H	-5.339285	-2.197848	-0.648124	H	1.583718	-1.180287	-2.881574
C	-2.049882	-0.223628	0.691066	C	3.419784	1.150945	-0.151852
H	-1.470261	0.488853	1.280337	H	4.135628	1.713944	0.449409
O	-1.196959	-1.361562	0.448047	O	2.496739	2.098322	-0.735299
N	-0.484727	-1.80505	1.536008	N	2.007085	3.085898	0.096254
O	-0.608492	-1.229036	2.592442	O	2.510848	3.225397	1.187649
O	0.20368	-2.764711	1.285951	O	1.125761	3.729989	-0.409964
C	-1.277814	1.551353	-0.974975	C	3.032981	0.328301	2.282466
H	-0.262917	1.184248	-0.826227	H	2.793177	1.340487	2.604545
H	-1.470288	2.354304	-0.258337	H	4.108209	0.159469	2.385515
H	-1.385414	1.941629	-1.98804	H	2.491822	-0.396375	2.892703
O	-2.091857	-0.439586	-1.769989	O	1.309977	0.332543	0.777787

Table S17: (α-pin-O_3−RO)$_2$[S14]

Atom	x	y	z	Atom	x	y	z
H	-3.615638	1.082673	2.490876	H	4.010904	-1.34618	2.249797
C	-2.874172	0.859417	1.72064	C	3.093374	-1.052485	1.735748
C	-2.740874	1.857053	0.550207	C	2.779822	-1.91016	0.485034
C	-3.226849	-0.240875	0.707649	C	3.278537	0.195516	0.85211
C	-2.632061	0.655905	-0.445986	C	3.255177	-0.728283	-0.428924
H	-1.911217	0.657091	2.198473	H	2.280756	-0.976951	2.462333
H	-4.304757	-0.356376	0.554431	H	4.228181	0.731088	0.95492
H	-3.694372	2.372642	0.377511	H	3.415693	-2.798035	0.378063
C	-1.188452	0.293079	-0.783995	C	2.355255	-0.314785	-1.587026
H	-0.748342	1.050626	-1.440134	H	2.334657	-1.09712	-2.35439
H	-1.133067	-0.668199	-1.305352	H	2.737055	0.596698	-2.057779
H	-0.571105	0.230131	0.116411	H	1.323902	-0.132249	-1.275857
C	-3.473743	0.760096	-1.709381	C	4.678182	-0.960105	-0.933049
H	-3.069261	1.531338	-2.376039	H	4.689087	-1.746283	-1.69664
H	-4.511314	1.0191	-1.474376	H	5.345634	-1.270222	-0.121505
H	-3.483006	-0.190044	-2.252238	H	5.081442	-0.043094	-1.374521
C	-1.607538	2.883274	0.641604	C	1.315136	-2.334256	0.39635
H	-1.869874	3.607086	1.426799	H	1.10755	-3.033613	1.220455
H	-0.664188	2.407285	0.923153	H	0.620653	-1.503072	0.539063
C	-1.427532	3.648144	-0.636217	C	0.975379	-3.072162	-0.866659
H	-2.316854	4.206302	-0.998438	H	1.789043	-3.699052	-1.291612
O	-0.400997	3.677963	-1.284404	O	-0.103001	-3.047148	-1.423106
C	-2.622901	-1.60313	0.907264	C	2.175511	1.21505	0.94075
C	-3.009856	-2.713061	-0.087289	C	2.342446	2.522472	0.155772
H	-2.977894	-3.695592	0.412471	H	2.125054	3.382358	0.814311
H	-2.150654	-2.724015	-0.801983	H	1.513107	2.574791	-0.590074
O	-1.82146	-1.865588	1.77926	O	1.169766	1.067594	1.609531
O	-4.119083	-2.494668	-0.818919	O	3.493497	2.67018	-0.536828

Table S18: MetO-EtO[S13]

Atom	x	y	z
C	-2.426812	0.316113	-0.000204
H	-2.984867	0.576073	-0.908457
H	-1.514383	0.949869	0.000292
H	-2.98507	0.574885	0.90826
O	-1.967637	-0.958568	-0.000984
H	3.041673	-0.026022	-0.883253
C	2.557931	-0.441143	0.000076
H	2.705025	-1.521432	-0.000192
H	3.041425	-0.026504	0.883767
C	1.077522	-0.108259	-0.000032
H	0.548536	-0.565499	-0.857031
H	0.54827	-0.566004	0.856525
O	0.768294	1.213373	0.000213

Table S19: MetO-ProO[S13]

Atom	x	y	z
C	2.579055	0.234229	0.340672
H	1.733961	0.330105	1.050703
H	2.742799	1.256338	-0.027871
H	3.451844	-0.144416	0.884022
O	2.137836	-0.593478	-0.638903
H	-1.357241	-1.959307	-0.523526
C	-1.066284	-1.336321	0.321224
H	-1.783037	-1.491466	1.128101
H	-0.079744	-1.646429	0.659515
C	-1.058154	0.139635	-0.087381
H	-0.275622	0.244168	-0.869782
C	-2.389765	0.622788	-0.653994
H	-3.174114	0.517945	0.096658
H	-2.673529	0.036569	-1.527976
H	-2.322633	1.670994	-0.939679
O	-0.567533	0.942077	0.898383

Table S20: MetO-AceO[S13]

Atom	x	y	z
C	-3.221839	-0.44167	-0.186535
H	-2.275896	-1.015157	-0.125608
H	-3.40081	-0.294096	-1.260104
H	-4.012631	-1.042056	0.277478
O	-2.969091	0.722866	0.460736
H	-0.68731	1.264468	-0.118438
C	0.395623	1.175356	-0.148494
H	0.780826	1.543219	-1.101448
H	0.861654	1.783141	0.626335
C	0.808807	-0.2532	-0.001507
C	2.330797	-0.517858	0.271385
H	2.351788	-0.411676	1.374843
H	2.558242	-1.557498	0.008308
O	0.07767	-1.199318	-0.066778
O	3.119261	0.410046	-0.272118

Table S21: MetO-ProOHO[S13]

Atom	x	y	z
C	2.75619	-0.70294	-0.185015
H	2.747014	-0.377051	-1.234884
H	1.850643	-1.338325	-0.101655
H	3.64791	-1.287064	0.057737
O	2.536388	0.337659	0.656508
H	-0.736142	0.988023	1.338736
C	-0.874337	1.039777	0.255923
H	-1.733315	1.672951	0.030427
C	-1.325832	-0.415881	-0.232736
H	-1.303148	-0.326395	-1.329347
C	-2.725392	-0.712135	0.292864
H	-3.454538	-0.021494	-0.132264
H	-2.750694	-0.630531	1.379969
H	-3.014241	-1.726244	0.021661
O	-0.381156	-1.248392	0.2171
O	0.223745	1.53604	-0.411475
H	1.03695	1.204624	-0.000005

Table S22: MetO-BuOHO[S13]

Atom	x	y	z
C	-2.897622	-1.209247	0.274354
H	-1.858282	-1.581976	0.166659
H	-3.608873	-2.006136	0.041323
H	-2.952441	-0.899265	1.327658
O	-2.97799	-0.141657	-0.558379
H	2.775308	-1.912086	-0.272456
C	3.22027	-0.996748	0.117032
H	3.282459	-1.088046	1.20289
H	4.235599	-0.920721	-0.270781
C	2.389382	0.216243	-0.281227
H	2.346308	0.299251	-1.369847
H	2.855575	1.128766	0.09815
C	0.962318	0.132443	0.257316
H	0.934509	0.195846	1.357228
C	0.139686	1.427351	-0.202121
H	0.002477	1.355549	-1.283659
H	0.806337	2.25991	0.024857
O	-1.039347	1.610173	0.486327
H	-1.743545	1.080485	0.081138
O	0.259298	-0.909384	-0.197199

Table S23: EtO-ProO[S13]

Atom	x	y	z
H	1.805315	1.531608	0.700584
C	2.610389	1.019632	0.174284
H	2.921719	1.63803	-0.66785
H	3.454814	0.912109	0.854485
C	2.141474	-0.342004	-0.313528
H	1.274719	-0.207304	-0.997327
H	2.896348	-0.852482	-0.932335
O	1.669817	-1.173912	0.646147
H	-2.810722	0.521605	1.592575
C	-2.513206	0.922263	0.622967
H	-3.35452	0.841858	-0.065678
H	-2.268842	1.976232	0.7487
C	-1.310727	0.160602	0.082998
H	-0.482548	0.16221	0.808729
C	-1.641551	-1.331142	-0.178699
H	-2.431191	-1.413197	-0.923672
H	-1.983177	-1.763649	0.760306
H	-0.758275	-1.868305	-0.514628
O	-0.875613	0.633675	-1.120103

Table S24: EtO-AceO[S13]

Atom	x	y	z
H	2.984295	-1.058052	1.352298
C	2.694035	-0.942483	0.308502
H	3.525791	-1.266811	-0.317031
H	1.838357	-1.583763	0.104463
C	2.348367	0.50671	0.013625
H	3.21984	1.175938	0.083486
H	2.014114	0.609173	-1.038978
O	1.308573	1.015013	0.723812
H	-0.608212	-2.294211	0.595333
C	-1.191058	-1.389745	0.743362
H	-2.245525	-1.629913	0.878962
H	-0.86383	-0.867033	1.642652
C	-1.032971	-0.480357	-0.433929
C	-1.754061	0.895865	-0.351686
H	-1.001512	1.486173	0.211059
H	-1.842009	1.318844	-1.360296
O	-0.382197	-0.729826	-1.410412
O	-2.880997	0.874964	0.36723

Table S25: EtO-ProOHO[S13]

Atom	x	y	z
H	-1.507375	-0.440471	1.441443
C	-2.497834	-0.607747	1.022437
H	-2.839176	-1.601302	1.311995
H	-3.188559	0.126981	1.434951
C	-2.453023	-0.498698	-0.493542
H	-1.713707	-1.231296	-0.881172
H	-3.40656	-0.756549	-0.974377
O	-1.970191	0.677137	-0.974106
H	1.273442	1.155238	-1.185876
C	1.331671	1.118811	-0.094946
H	2.210684	1.67127	0.239144
C	1.642922	-0.396598	0.309969
H	1.555974	-0.381432	1.407365
C	3.048116	-0.76568	-0.150338
H	3.797336	-0.163771	0.365275
H	3.150073	-0.61444	-1.225492
H	3.243412	-1.814984	0.06501
O	0.668133	-1.118675	-0.25189
O	0.220368	1.643621	0.527361
H	-0.576241	1.376384	0.043259

Table S26: EtO-BuOHO[S13]

Atom	x	y	z
H	1.76058	-0.646321	-1.41962
C	2.697716	-1.014389	-1.006322
H	2.801778	-2.07013	-1.255502
H	3.525681	-0.470659	-1.460107
C	2.712655	-0.836619	0.503808
H	1.837437	-1.369111	0.932747
H	3.595483	-1.281384	0.983093
O	2.514424	0.435729	0.937713
H	-2.849219	-2.05174	0.440612
C	-3.383392	-1.246868	-0.063838
H	-3.384871	-1.462781	-1.13374
H	-4.416981	-1.256754	0.280701
C	-2.723173	0.096177	0.220104
H	-2.741168	0.302556	1.29296
H	-3.277443	0.896688	-0.275871
C	-1.272801	0.131597	-0.257583
H	-1.202431	0.073344	-1.356299
C	-0.631614	1.55911	0.071663
H	-0.548099	1.632526	1.158825
H	-1.372554	2.275635	-0.284152
O	0.557561	1.797354	-0.582276
H	1.284012	1.382218	-0.09219
O	-0.469884	-0.758394	0.33421

Table S27: ProO-AceO[S13]

Atom	x	y	z
H	4.034159	-0.593863	0.481895
C	3.381424	-0.613672	-0.390374
H	3.820203	0.022877	-1.159515
H	3.33039	-1.632133	-0.770678
C	1.98694	-0.108193	-0.030494
H	1.565761	-0.768537	0.758628
C	1.971277	1.314747	0.530125
H	2.297786	2.017612	-0.236959
H	2.649667	1.393629	1.378691
H	0.970294	1.58232	0.862324
O	1.080561	-0.285771	-1.032573
H	-1.52214	-1.655957	-1.070069
C	-1.927756	-1.61409	-0.058703
H	-3.013646	-1.636574	-0.153485
H	-1.578708	-2.457246	0.530104
C	-1.512456	-0.330701	0.588953
C	-1.824665	0.972419	-0.189005
H	-1.782029	1.830212	0.495386
H	-0.931527	1.015229	-0.850971
O	-0.936379	-0.250353	1.639764
O	-2.905757	0.912056	-0.971098

Table S28: ProO-ProOHO[S13]

Atom	x	y	z
H	3.677535	-1.25316	-0.750727
C	3.401998	-0.207907	-0.613482
H	4.08541	0.232685	0.113462
H	3.519319	0.314214	-1.561144
C	1.966562	-0.093297	-0.116653
H	1.27845	-0.557112	-0.860313
C	1.702604	-0.822564	1.203678
H	2.306189	-0.381852	1.997607
H	1.970898	-1.873565	1.107805
H	0.651246	-0.750583	1.47373
O	1.500528	1.184879	-0.109347
H	-2.945762	1.464288	0.061392
C	-1.937873	1.143604	-0.205772
H	-1.600451	1.690114	-1.088076
C	-2.125238	-0.389582	-0.640402
H	-2.934534	-0.328994	-1.387334
C	-2.535135	-1.231936	0.560826
H	-3.488774	-0.88476	0.960211
H	-1.78339	-1.165711	1.344319
H	-2.645044	-2.272857	0.26037
O	-0.963582	-0.744428	-1.199211
O	-1.119097	1.323654	0.88819
H	-0.191673	1.331916	0.600676

Table S29: ProO-BuOHO[S13]

Atom	x	y	z
H 3.765402	1.204354	1.186819	
C 3.640471	0.260692	0.656323	
H 4.399853	0.204756	-0.124809	
H 3.799618	-0.558447	1.354948	
C 2.250323	0.171035	0.041453	
H 1.481154	0.237878	0.849396	
C 1.927772	1.3077	-0.935202	
H 2.625322	1.283704	-1.77294	
H 2.024949	2.268628	-0.432144	
H 0.913854	1.209081	-1.318003	
O 1.961784	-1.053547	-0.470744	
H -4.188301	0.537982	0.183507	
C -3.40348	0.968375	-0.442217	
H -3.319998	0.365452	-1.346124	
H -3.730727	1.963658	-0.740221	
C -2.078394	1.040287	0.303381	
H -2.14492	1.770865	1.111363	
H -1.281236	1.367507	-0.363331	
C -1.658853	-0.288597	0.935414	
H -2.470418	-0.709682	1.554858	
C -1.447594	-1.420521	-0.184458	
H -2.457346	-1.654987	-0.52171	
H -1.022736	-2.291348	0.316599	
O -0.710416	-1.004605	-1.272545	
H 0.23878	-1.081139	-1.079228	
O -0.503224	-0.236182	1.604945	

Table S30: AceO-ProOHO[S13]

Atom	x	y	z
H	2.528577	0.829228	2.106196
C	1.884999	0.280287	1.425265
H	1.755987	-0.752505	1.748876
H	0.896313	0.739155	1.36697
C	2.473587	0.277989	0.054472
C	1.599713	-0.501646	-1.075035
H	0.898878	0.324661	-1.304024
H	2.297299	-0.701101	-1.894362
O	3.476419	0.801994	-0.296516
O	1.006546	-1.552751	-0.555669
H	-3.024512	-0.678261	1.356375
C	-2.009666	-0.505323	0.996206
H	-1.342212	-0.335327	1.844038
C	-2.122056	0.901021	0.202561
H	-2.613297	1.548049	0.948625
C	-2.97323	0.725891	-1.047037
H	-3.977646	0.394984	-0.781136
H	-2.524195	-0.012114	-1.708065
H	-3.050212	1.676357	-1.573132
O	-0.861229	1.267239	-0.021841
O	-1.640991	-1.564563	0.208325
H	-0.684425	-1.540357	0.028956

Table S31: AceO-BuOHO[S13]

Atom	x	y	z
H	1.654863	-1.998768	1.590438
C	1.491923	-1.011023	1.169787
H	0.485729	-0.911946	0.760646
H	1.611863	-0.238833	1.930192
C	2.470864	-0.752372	0.075715
C	2.250195	0.644817	-0.731665
H	3.191854	0.839331	-1.254296
H	1.460853	0.273686	-1.413997
O	3.354997	-1.453741	-0.284349
O	1.833805	1.590321	0.078921
H	-2.057384	-2.502635	-0.404655
C	-2.93972	-1.968176	-0.051627
H	-3.649489	-1.914009	-0.879025
H	-3.397358	-2.556738	0.742916
C	-2.56629	-0.577719	0.445134
H	-1.861394	-0.64065	1.274447
H	-3.452879	-0.059815	0.818606
C	-1.943226	0.271857	-0.660816
H	-2.680078	0.512143	-1.447727
C	-1.562965	1.732938	-0.083633
H	-2.530324	2.158206	0.187451
H	-1.127003	2.295201	-0.91092
O	-0.760723	1.698041	1.030783
H	0.170075	1.648561	0.749313
O	-0.807047	-0.203237	-1.165689

Non-Ergodicity of Bimolecular Complexes

The possible non-ergodicity of bimolecular complexes impacts the accuracy of the typical models we might use to model the dissociation. In this section we will discuss the applicability of the detailed balance and ergodicity assumptions in terms of the triplet state $(\mathrm{MetO})_2$ dimer, the simplest bimolecular complex we are interested in. The detailed balance assumption of association and dissociation holds if the equilibrium energy distribution (Boltzmann distribution) of the dissociating system and the steady-state energy distribution do not differ significantly:[S15]

$$\int_0^\infty \frac{\rho_{ss}^2(E)}{\rho_{eq}(E)} \mathrm{d}E \approx 1 \tag{26}$$

The cited paper formulates the same argument more rigorously in terms of energy relaxation eigenvectors of the master equation matrix, but the substance of the argument stays the same: Detailed balance breaks when the steady-state energy distribution differs significantly from the Boltzmann distribution, and the important physical parameters for determining this is the ratio between the chemically significant eigenvalue (the dissociation rate) and the lowest energy relaxation eigenvalues (the slowest rates). If the former sits firmly outside the numerical continuum of the latter group, then dissociation rates calculated using Equation 2 are reliable.

If the dissociation *outspeeds* the intermolecular energy relaxation, than the complex is non-ergodic, in which case kinetic methodologies derived using statistical ensembles (The Eyring equation, RRKM, etc.) are all inaccurate, at least if the the complex as a whole is treated as a system in equilibrium. It has long been well-known that dissociation reactions with weakly bound complexes have this property, complexes with noble gas bonds being a particularly strong example.[S16] Marcus himself made a distinction between weakly and

strongly bound complexes when discussing the applicability of the RRKM model for dissociation reactions.[S17]

All of this depends on the magnitude of the anharmonic couplings. Energy flows from one oscillator to another due to the existence of non-linear resonances, which depends on the magnitude of the anharmonic couplings. We investigate this by performing an automated VPT2[S18] anharmonicity analysis of the $(MetO)_2$ vibrational modes using ωB97XD/aug-cc-pVTZ,[S5,S19] the same level of quantum theory that the geometry was optimized with in Source.[S11] This calculation was performed in the Gaussian 16 program suite.[S4] The total anharmonic energy, excluding the rovibrational coupling is thus described by the series expression (in mass-weighted atomic units):

$$V_{AnH} = \frac{1}{2}\sum_{i}^{N} K_{ii}q_i^2 + \frac{1}{6}\sum_{i,j,k}^{\frac{(N+2)!}{3!(N-1)!}} K_{ijk}q_iq_jq_k + \frac{1}{24}\sum_{i,j,k,l}^{\frac{(N+2)!}{4!(N-2)!}} K_{ijkl}q_iq_jq_kq_l \qquad (27)$$

where K are derivatives of the potential energy. $K_{ii} = \left(\frac{\partial^2 V}{\partial q_i^2}\right)$ are the harmonic force constants, whereas $K_{ijk} = \left(\frac{\partial^3 V}{\partial q_i \partial q_j \partial q_k}\right)$ and $K_{ijkl} = \left(\frac{\partial^4 V}{\partial q_i \partial q_j \partial q_k \partial q_l}\right)$ are the cubic and quartic anharmonic coupling constants. The 24 internal degrees of freedom of $(MetO)_2$ were all characterized as either intramolecular or intermolecular. This may be a bit theoretically sloppy, as the VPT2 calculation was performed in a normal mode basis in which all vibrations are in principle oscillations of the system as a whole. However, inspection of the vibrational eigenvectors (visualised in Figures S35, S36 and S37 along with the vibrational frequencies in cm^{-1}.) reveals that the vibrational motion is indeed *mainly* localized in either of the two radicals for the 18 modes characterized as intramolecular. The six modes characterized as intermolecular are recognizably the six intermolecular bounded translations and torsions referred to in our 'Equations of Motion' section.

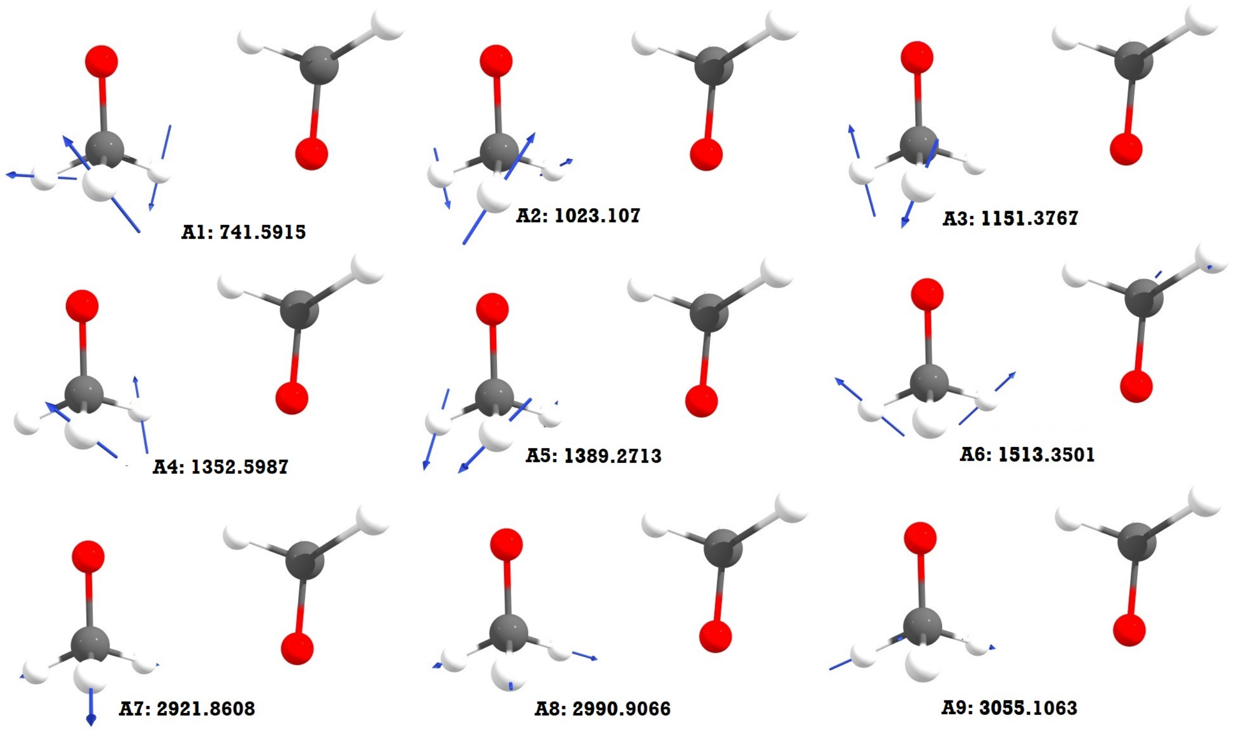

Figure S35: Visualisation of the nine intramolecular modes in 'Molecule A'. The vectors on individual atoms are scaled by magnitude.

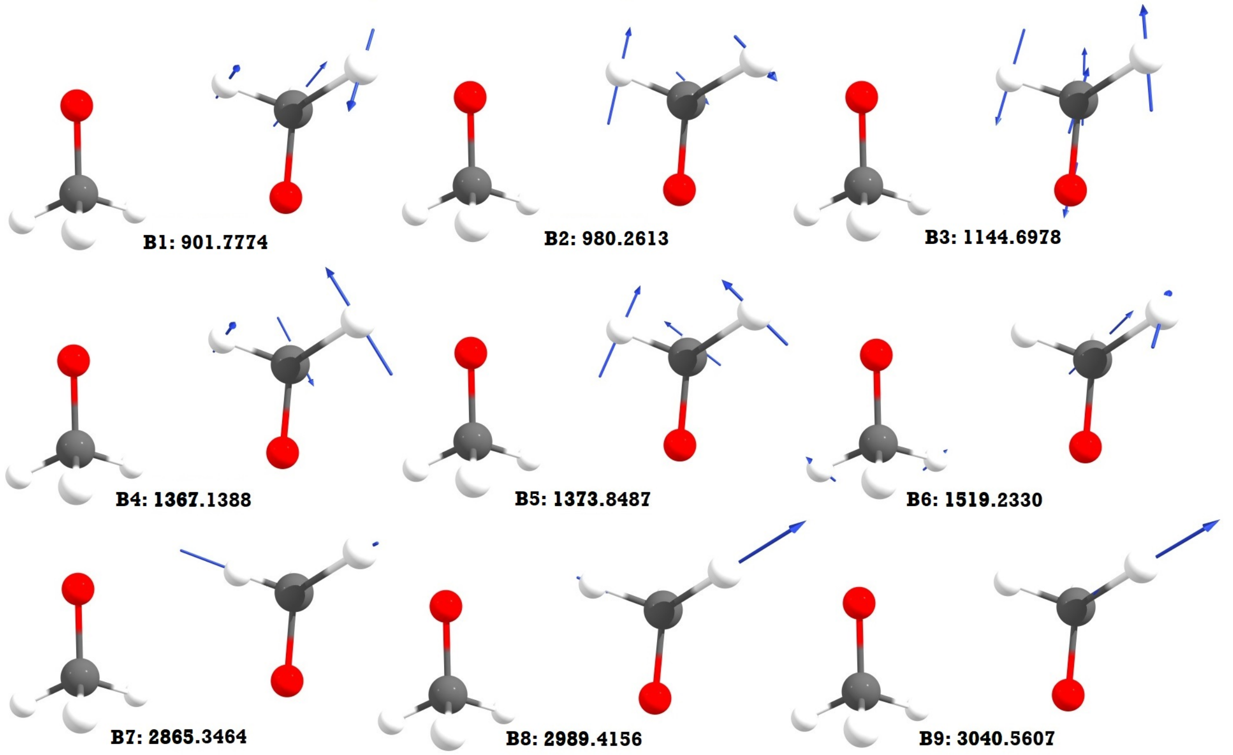

Figure S36: Visualisation of the nine intramolecular modes in 'Molecule B'.

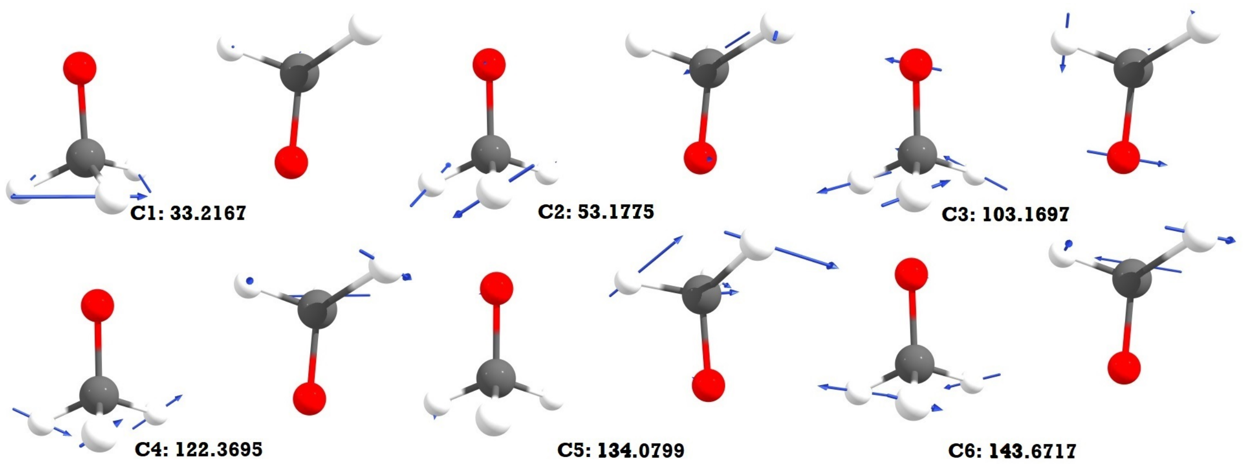

Figure S37: Visualisation of the six low-frequency intermolecular modes.

A set of completely harmonic oscillators are orthogonal in the normal mode basis, which means that there are no crossings in their phase space trajectories, rendering energy exchange is impossible. The rate of vibrational energy relaxation thus depends on the existence of non-linear resonances between the modes, whose locations in phase space depends on the anharmonic couplings. Thus all the cubic coupling constants from the VPT2 calculation were categorized based on if they are intramolecular or intermolecular, and the coupling strengths averaged for each category. The strongest coupling constants in each category are presented in Tables S32-S36. The precise dynamics of these coupled systems is highly complex,[S20] and exact quantum mechanical solutions exist only for model systems.[S21,S22] One useful model system for our case, however, is a recent study by Zhang et.al,[S23] focusing on modelling a 'ergodicity phase diagram' for a system composed of two fragments interacting via a Bose Statistics Triangle Rule potential,[S24] in which the cubic anharmonic couplings are expressed as $\frac{1}{6}\sum_{i,j,k}^{\frac{(N+2)!}{3!(N-1)!}} K_{ijk}q_iq_jq_k = -V_{rx}\prod_i \left(\frac{\omega_i}{\Omega}\right)^{\frac{3}{2}} a^3$. Here V_{rx} 'is on the order of a typical anharmonicity constant $\tilde{\chi}_{ij}$', which in our case seems to be on average 77 cm^{-1} for the intermolecular couplings (Table S36). The a parameter here describes the ratio of higher-order coupling terms compared to the lower-order terms. It it typically between 0.1 and 0.3 depending on the 'softness' of the vibrational modes, coupled methyl rotations being on the softer end of the scale. In Fig. 4 of Source S23, a 'ergodicity phase diagram' is presented

for various coupling strengths. If the coupling potential V_{rx} is taken as the strength of the intermolecular modes in our case, we are located in the region $\frac{V_{rx}}{\Omega} = \frac{77\ \mathrm{cm}^{-1}}{1390\ \mathrm{cm}^{-1}} \leq 0.06$ of the diagram, in which even partial ergodicity requires a very 'soft' coupling of $a \geq 0.25$. Somewhat problematically, the modelled diagrams all have higher excitation energies than our complexes would realistically have, as the 'low' excitation energy of 2000 cm^{-1} exceeds the dissociation energy of $(\mathrm{MetO})_2$ by over 70 %. Thus we may conclude that the white 'non-ergodic' area of the diagram will be further shifted to the right, requiring exceptionally strong coupling for ergodicity to apply. As seen Tables S32-S36, the coupling between the torsional modes of the $-\mathrm{CH}_3$ groups and the stretching of the CH bonds is quite strong, and these may or may not provide the necessary energy flow for partial ergodicity. If this is the case, it should be noted that this is due to the proximity of the two methyl groups, a property the $(\mathrm{MetO})_2$ complex doesn't share with many other alkoxyl radical complexes. Thus we may conclude that dissociation of the complex at the very least poses a serious competition for internal energy relaxation, and that equilibrium models for calculating the dissociation rate like Detailed Balance or RRKM may overrestimate the amount of available energy the dissociative modes have at their disposal.

Table S32: The 40 strongest coupling constants internal to Molecule A.

i	j	k	$\tilde{\chi}_{ijk}$ (cm^{-1})	K_{ijk} $\left(\frac{\text{Ha}}{\text{Bohr}^{-3}\text{Amu}^{-\frac{3}{2}}}\right)$
A8	A9	A9	-884.50846	-0.50969
A7	A8	A8	-787.47393	-0.43908
A7	A9	A9	-588.2855	-0.33506
A8	A8	A9	-441.46759	-0.25171
A7	A7	A8	454.74992	0.25062
A7	A7	A9	-156.09752	-0.08695
A1	A1	A7	487.33596	0.06738
A6	A9	A9	123.76654	0.05073
A1	A6	A9	-219.02181	-0.04423
A1	A4	A8	-221.37774	-0.04182
A2	A7	A7	-126.22192	-0.04086
A4	A8	A8	101.28423	0.03842
A2	A8	A8	-114.75523	-0.03803
A2	A6	A9	147.09196	0.03504
A2	A3	A7	-159.95744	-0.03251
A6	A6	A8	112.89795	0.03223
A2	A5	A9	-140.80482	-0.03214
A3	A9	A9	-88.5675	-0.03167
A5	A8	A9	81.09019	0.03151
A4	A4	A8	122.71395	0.03131
A5	A5	A9	-114.70097	-0.03038
A2	A3	A3	221.69422	0.02828
A1	A5	A9	-143.3861	-0.02774
A1	A1	A3	-317.97192	-0.0276
A7	A8	A9	48.53029	0.02735
A2	A5	A8	120.3343	0.02718
A1	A1	A9	-190.9847	-0.027
A1	A8	A9	94.44277	0.02681
A5	A5	A7	102.65893	0.02659
A3	A3	A7	123.25808	0.02646
A2	A2	A7	132.24742	0.02545
A4	A4	A7	100.15972	0.02526
A3	A8	A8	-70.12597	-0.02455
A1	A2	A3	238.57911	0.02443
A3	A6	A9	-94.35246	-0.02374
A3	A7	A7	-64.68223	-0.02212
A1	A3	A8	125.47695	0.02187
A1	A1	A4	-229.57636	-0.0216
A1	A7	A7	-77.67622	-0.02132
A1	A2	A2	-215.18176	-0.02086
⋮	⋮	⋮	⋮	⋮
		$\frac{1}{156}\sum\lvert\mathbf{X_{ijk}}\rvert$	**81.17407**	**0.02426**

Table S33: The 40 strongest coupling constants internal to Molecule B.

i	j	k	$\tilde{\chi}_{ijk}$ (cm^{-1})	K_{ijk} $\left(\frac{Ha}{Bohr^{-3}Amu^{-\frac{3}{2}}}\right)$
B8	B9	B9	-1347.51471	-0.77261
B7	B8	B8	473.18607	0.26115
B7	B7	B8	430.67322	0.2327
B7	B9	B9	360.22561	0.20221
B1	B1	B7	-480.61978	-0.08001
B1	B1	B2	-744.64673	-0.07251
B2	B7	B7	194.68583	0.06024
B1	B6	B9	-259.41058	-0.05774
B6	B9	B9	-139.82422	-0.05715
B1	B1	B3	-469.97087	-0.04945
B2	B5	B8	-220.57813	-0.04827
B2	B4	B9	-190.12502	-0.04186
B4	B8	B9	-102.35677	-0.03935
B6	B6	B8	132.26275	0.03789
B2	B3	B7	-179.3072	-0.03507
B3	B9	B9	-96.64991	-0.03429
B1	B4	B8	-161.1215	-0.03374
B1	B8	B9	-107.91321	-0.0337
B2	B2	B8	173.21704	0.03202
B3	B8	B8	-91.64639	-0.03197
B2	B2	B7	-169.88802	-0.03074
B3	B7	B7	-89.60363	-0.02996
B5	B5	B8	110.85461	0.02872
B1	B4	B7	-128.81298	-0.0264
B4	B5	B9	101.14399	0.02636
B4	B4	B8	98.70718	0.02545
B1	B3	B4	-174.73083	-0.02264
B5	B8	B8	-59.00749	-0.02255
B5	B5	B7	-85.86563	-0.02178
B2	B5	B7	-99.4558	-0.02131
B3	B5	B7	-89.02488	-0.02061
B5	B7	B8	-50.24704	-0.0188
B4	B4	B7	-72.03204	-0.01818
B2	B7	B8	57.53339	0.01818
B2	B3	B3	-145.3203	-0.01796
B1	B1	B6	-136.4718	-0.01654
B3	B3	B7	-75.38975	-0.01593
B4	B4	B6	-83.79309	-0.0154
B2	B8	B8	47.084	0.0152
B4	B7	B9	-37.12884	-0.01398
⋮	⋮	⋮	⋮	⋮
		$\frac{1}{156}\sum\lvert\mathbf{X_{ijk}}\rvert$	**65.30468**	**0.019270**

Table S34: The 18 strongest intermolecular couplings between the intramolecular modes.

i	j	k	$\tilde{\chi}_{ijk}$ (cm^{-1})	K_{ijk} $\left(\frac{Ha}{Bohr^{-3}Amu^{-\frac{3}{2}}}\right)$
A8	B9	B9	80.56509	0.0462
B8	A8	A8	-71.35385	-0.04024
B8	B8	A8	63.76371	0.03595
B8	A9	A9	-59.85135	-0.03448
A7	B8	A8	-49.83228	-0.02778
B7	B7	A8	-45.7305	-0.02472
B9	A9	A9	-41.88717	-0.02434
A6	B9	B9	59.40514	0.02423
B8	B9	A9	-41.77675	-0.02401
B1	A6	B9	103.4997	0.02299
B6	A9	A9	51.67964	0.02122
B7	B8	A8	-35.13684	-0.0194
A1	B6	A9	-90.63616	-0.01834
A8	B9	A9	29.43356	0.01692
A7	A7	B8	28.95736	0.01595
B8	A8	A9	-26.20081	-0.01493
B7	A9	A9	-25.7855	-0.01454
A2	B6	A9	60.46939	0.01443
⋮	⋮	⋮	⋮	⋮
		$\frac{1}{810}\sum\lvert\mathbf{X_{ijk}}\rvert$	**7.98907**	**0.001909**

Table S35: The 12 strongest couplings between the six intermolecular modes.

i	j	k	$\tilde{\chi}_{ijk}$ (cm^{-1})	K_{ijk} $\left(\frac{Ha}{Bohr^{-3}Amu^{-\frac{3}{2}}}\right)$
C1	C3	C6	91.45874	0.00022
C1	C6	C6	75.82293	0.00022
C3	C4	C4	-43.60605	-0.00019
C4	C4	C6	-36.58253	-0.00019
C1	C3	C3	85.84785	0.00018
C3	C6	C6	35.26407	0.00018
C1	C1	C6	119.17692	0.00016
C1	C1	C3	127.01037	0.00015
C3	C4	C5	29.09915	0.00013
C3	C3	C6	27.18556	0.00012
C4	C6	C6	21.44134	0.00012
C1	C4	C6	40.15453	0.00011
⋮	⋮	⋮	⋮	⋮
		$\frac{1}{60}\sum\lvert\mathbf{X_{ijk}}\rvert$	**20.34691**	**0.00005**

Table S36: The 40 strongest couplings including the six intermolecular modes.

i	j	k	$\tilde{\chi}_{ijk}$ (cm^{-1})	K_{ijk} $\left(\frac{Ha}{Bohr^{-3}Amu^{-\frac{3}{2}}}\right)$
C1	C1	A7	9330.64844	0.05778
C1	A4	A8	1063.83864	0.04253
C1	A1	A9	-1172.93373	-0.03509
C1	A5	A9	-807.37004	-0.03306
C6	B5	B9	-362.78629	-0.03065
C4	B1	B8	470.82942	0.02949
C6	B1	B8	-430.91622	-0.02925
C4	B5	B9	374.4757	0.0292
C1	A2	A8	-807.7542	-0.02821
C6	C6	B7	-1062.32037	-0.02818
C6	B2	B9	392.92828	0.02804
C2	B1	B8	664.32301	0.02743
C2	B6	B9	-506.5785	-0.02738
C4	C6	B7	1114.74097	0.02729
C1	C6	A7	2096.6839	0.027
C6	B6	B9	299.54602	0.02661
C4	C4	B7	-1175.71368	-0.02656
C4	B2	B9	-401.82748	-0.02647
C4	B6	B9	-321.31854	-0.02635
C6	B4	B8	-308.19134	-0.02576
C6	A1	A8	414.25181	0.0255
C6	C6	B8	914.7568	0.02478
C6	B1	B7	-367.79807	-0.02444
C1	A1	A7	-828.88869	-0.02425
C4	B4	B8	313.86605	0.02421
C6	A6	A9	-269.27137	-0.02394
C4	B1	B7	383.50585	0.02352
C4	C4	B8	1014.50603	0.02341
C4	C6	B8	-923.77603	-0.0231
C1	A1	A8	758.34087	0.02245
C2	A5	A9	-419.9864	-0.02176
C1	C3	A7	1904.15558	0.02078
C4	A6	A9	-252.59272	-0.02072
C6	A2	A8	-271.69134	-0.01973
C1	C1	A8	3006.18301	0.01883
C1	A6	A9	-439.39867	-0.01878
C1	A4	A9	447.55945	0.01808
C6	B4	B7	-220.68011	-0.01806
C1	C2	A7	2278.21707	0.01785
C4	B4	B7	235.67515	0.0178
⋮	⋮	⋮	⋮	⋮
		$\frac{1}{1394}\sum \lvert \mathbf{X_{ijk}} \rvert$	**76.77464**	**0.002045**

References

(S1) Landau, L. D.; Lifshitz, E. M. *Course of Theoretical Physics Volume 5, Statistical Physics Part 1, 3th Revised English Edition*; Elsevier, 1980; pp 257–262.

(S2) Landau, L. D.; Lifshitz, E. M. *Course of Theoretical Physics Volume 2, The Classical Theory of Fields, 4th Revised English Edition*; Pergamon Press, 1975; pp 96–99.

(S3) Tang, K. T.; Toennies, J. P. An improved simple model for the van der Waals potential based on universal damping functions for the dispersion coefficients. *J. Chem. Phys.* **1984**, *80*, 3726–3741.

(S4) Frisch, M. J.; Trucks, G. W.; Schlegel, H. B.; Scuseria, G. E.; Robb, M. A.; Cheeseman, J. R.; Scalmani, G.; Barone, V.; Petersson, G. A.; Nakatsuji, H.; Li, X.; Caricato, M.; Marenich, A. V.; Bloino, J.; Janesko, B. G.; Gomperts, R.; Mennucci, B.; Hratchian, H. P.; Ortiz, J. V.; Izmaylov, A. F.; Sonnenberg, J. L.; Williams-Young, D.; Ding, F.; Lipparini, F.; Egidi, F.; Goings, J.; Peng, B.; Petrone, A.; Henderson, T.; Ranasinghe, D.; Zakrzewski, V. G.; Gao, J.; Rega, N.; Zheng, G.; Liang, W.; Hada, M.; Ehara, M.; Toyota, K.; Fukuda, R.; Hasegawa, J.; Ishida, M.; Nakajima, T.; Honda, Y.; Kitao, O.; Nakai, H.; Vreven, T.; Throssell, K.; Montgomery, J. A., Jr.; Peralta, J. E.; Ogliaro, F.; Bearpark, M. J.; Heyd, J. J.; Brothers, E. N.; Kudin, K. N.; Staroverov, V. N.; Keith, T. A.; Kobayashi, R.; Normand, J.; Raghavachari, K.; Rendell, A. P.; Burant, J. C.; Iyengar, S. S.; Tomasi, J.; Cossi, M.; Millam, J. M.; Klene, M.; Adamo, C.; Cammi, R.; Ochterski, J. W.; Martin, R. L.; Morokuma, K.; Farkas, O.; Foresman, J. B.; Fox, D. J. Gaussian˜16 Revision C.01. 2016; Gaussian Inc. Wallingford CT.

(S5) Chai, J.-D.; Head-Gordon, M. Long-range corrected hybrid density functionals with damped atom–atom dispersion corrections. *Phys. Chem. Chem. Phys.* **2008**, *10*, 6615–6620.

(S6) Papajak, E.; Zheng, J.; Xu, X.; Leverentz, H. R.; Truhlar, D. G. Perspectives on Basis Sets Beautiful: Seasonal Plantings of Diffuse Basis Functions. *J. Chem. Theory Comput.* **2011**, *7*, 3027–3034.

(S7) Strekalov, M. Partition function of the hindered rotor: Analytical solutions. *Chem. Phys.* **2009**, *355*, 62–66.

(S8) Andrews, S. S. Using Rotational Averaging To Calculate the Bulk Response of Isotropic and Anisotropic Samples from Molecular Parameters. *Journal of Chemical Education* **2004**, *81*, 877.

(S9) Elm, J.; Kubečka, J.; Besel, V.; Jääskeläinen, M. J.; Halonen, R.; Kurten, T.; Vehkamäki, H. Modeling the formation and growth of atmospheric molecular clusters: A review. *J. Aerosol Sci.* **2020**, *149*, 105621.

(S10) Ray, B. S. Über die Eigenwerte des asymmetrischen Kreisels. *Z. Physik* **1932**, *78*, 74–91.

(S11) Hasan, G.; Salo, V.-T.; Valiev, R. R.; Kubečka, J.; Kurtén, T. Comparing Reaction Routes for ^{3}(RO···OR) Intermediates Formed in Peroxy Radical Self- and Cross-Reactions. *J. Phys. Chem. A* **2020**, *124*, 8305–8320.

(S12) Hasan, G.; Valiev, R. R.; Salo, V.-T.; Kurtén, T. Computational Investigation of the Formation of Peroxide (ROOR) Accretion Products in the OH- and NO3-Initiated Oxidation of α-Pinene. *J. Phys. Chem. A* **2021**, *125*, 10632–10639.

(S13) Hasan, G.; Salo, V.-T.; Golin Almeida, T.; Valiev, R. R.; Kurtén, T. Computational Investigation of Substituent Effects on the Alcohol + Carbonyl Channel of Peroxy Radical Self- and Cross-Reactions. *J. Phys. Chem. A* **2023**, *127*, 1686–1696.

(S14) Peräkylä, O. J.; Berndt, T.; Franzon, L.; Hasan, G.; Meder, M.; Valiev, R.; Daub, C.; Varelas, J. G.; Geiger, F. M.; Thomson, R. J.; Rissanen, M. P.; Kurtén, T.; Ehn, M. K.

A large gas-phase source of esters and other accretion products in the atmosphere. *J. Am. Chem. Soc.* **2023**, Accepted for Publication.

(S15) Miller, J. A.; Klippenstein, S. J. Some observations concerning detailed balance in association/dissociation reactions. *J. Phys. Chem. A* **2004**, *108*, 8296–8306.

(S16) Levine, R. D.; Bernstein, R. B. *Molecular reaction dynamics and chemical reactivity*; Oxford University Press, USA, 1987; pp 426–444.

(S17) Marcus, R. A. Unimolecular dissociations and free radical recombination reactions. *J. Chem. Phys.* **1952**, *20*, 359–364.

(S18) Bloino, J.; Biczysko, M.; Barone, V. General perturbative approach for spectroscopy, thermodynamics, and kinetics: methodological background and benchmark studies. *J. Chem. Theory Comput.* **2012**, *8*, 1015–1036.

(S19) Kendall, R. A.; Dunning Jr, T. H.; Harrison, R. J. Electron affinities of the first-row atoms revisited. Systematic basis sets and wave functions. *J. Chem. Phys.* **1992**, *96*, 6796–6806.

(S20) Chirikov, B. V. A universal instability of many-dimensional oscillator systems. *Phys. Rep.* **1979**, *52*, 263–379.

(S21) Uzer, T.; Miller, W. Theories of intramolecular vibrational energy transfer. *Phys. Rep.* **1991**, *199*, 73–146.

(S22) Atkins, K. M.; Logan, D. E. Intersecting resonances as a route to chaos: classical and quantum studies of a three-oscillator model. *Phys. Lett. A* **1992**, *162*, 255–262.

(S23) Zhang, C.; Sibert III, E. L.; Gruebele, M. A phase diagram for energy flow-limited reactivity. *J. Chem. Phys.* **2021**, *154*, 104301.

(S24) Gruebele, M. Bose statistics triangle rule model for intramolecular vibrational energy redistribution. *J. Phys. Chem.* **1996**, *100*, 12183–12192.
