# Supplementary material for: Simple Physical Model for the Estimation of Irreversible Dissociation Rates for Bimolecular Complexes
Source: J Phys Chem A. 2023 Jul 7;127(28):5956–66. doi: 10.1021/acs.jpca.3c01890 (PMC10364079; doi:10.1021/acs.jpca.3c01890)
Supplement: Supplementary file 2 — jp3c01890_si_002.zip [file jp3c01890_si_002.zip › Supplementary/Readme.pdf]

Supporting Information: Simple physical model for estimation of irreversible dissociation rates for bimolecular complexes

Lauri Franzon*

Department of Chemistry, University of Helsinki,

P.O. Box 55 (A.I. Virtasen aukio 1), 00014 Helsinki, Finland

E-mail: lauri.franzon@helsinki.fi

Table of Contents

A description of the contents of each directory:

- **Code** The code for simulating the trajectories and calculating the dissociation rates. The contents in the **Bash&Py** and **Jupyter Notebook** directories are equivalent. See the separate instruction file in the directory on how to run the code.

- **Output Files** The various output files of the code for the model alkoxyl radical complexes covered in the article.

  - **Distance var** All output files for the simulations with variable cutoff distances, whose final results are presented in Table S1.

  - **Fit Files** Output files of the curve and surface fit modules of the code, including final values and confidence intervals for all fit parameters and fit R^2 coefficients.

  - **Parameter files** The files including the saved parameters D, μ, v_c, $\frac{\mu}{2k}$ ('v-coef'), $\langle l^2 \rangle$, $v_c(\langle l^2 \rangle)$, α, β, Θ, and maximum velocity to simulate ('vlim') for each complex. See Tables S3-S6 for the significance of these parameters.

  - **PES Shape** All output files for the PES surface shape related trajectories, whose final results are presented in Table S2.

  - **Results** The escape time $t(r_e \rightarrow 3r_e)$ for each trajectory (v, l) for each complex.

- **PES Scans** Gaussian 16 output files of the PES scans performed for the benchmarking RRKM calculations, as well as for the section '(In)accuracy of Lennard Jones Potential'. The details of these calculations are described in that section.

- **Freqs&Rots** Vibrational frequencies and rotational constants for the RRKM calculations, extracted from the PES Scan results with a script. The respective directories also include RRKM raw data in text files.
